# GLP-1 Induces the Expression of FNDC5 Derivatives That Execute Lipolytic Actions

**DOI:** 10.3389/fcell.2021.777026

**Published:** 2021-11-11

**Authors:** Hui Li, William Donelan, Fang Wang, Peilan Zhang, Lijun Yang, Yousong Ding, Dongqi Tang, Shiwu Li

**Affiliations:** ^1^ Center for Gene and Immunotherapy, The Second Hospital, Cheeloo College of Medicine, Shandong University, Jinan, China; ^2^ Department of Pathology, Immunology and Laboratory Medicine, College of Medicine, University of Florida, Gainesville, FL, United States; ^3^ Department of Urology, College of Medicine, University of Florida, Gainesville, FL, United States; ^4^ Institute of Medical Sciences, The Second Hospital, Cheeloo College of Medicine, Shandong University, Jinan, China; ^5^ Department of Medicinal Chemistry, Center for Natural Products, Drug Discovery, and Development, College of Pharmacy, University of Florida, Gainesville, FL, United States

**Keywords:** GLP-1, FNDC5, lipolysis, β-cells, obesity

## Abstract

Multiple GLP-1-derived therapeutics are clinically used to treat type 2 diabetes and obesity. However, the underlying mechanism of how these drugs regulate the body weight of obese patients remains incompletely understood. Here, we report that the lipolysis effects of GLP-1 on β cells can depend on its induced expression of fibronectin type III domain containing 5 (FNDC5). The transmembrane FNDC5 is a precursor of the recently identified hormone irisin that possesses a range of bioactivities, including anti-obesity and anti-diabetes. We revealed that GLP-1 upregulates the expression and secretion of FNDC5 in β cells, while GLP-1 itself fails to activate the lipolysis genes in FNDC5-knockout β cells. In addition, liraglutide, a clinically used GLP-1 receptor agonist, induced the expression of FNDC5 in mouse pancreas and brain tissues and increased the serum level of secreted FNDC5. Furthermore, we observed the expression of the well-known membrane-associated FNDC5 and a novel, secretable FNDC5 (sFNDC5) isoform in β cells and multiple rat tissues. Recombinant sFNDC5 stimulated lipolysis of wild type and FNDC5-knockout β cells. This new isoform further induced lipolysis and browning of adipocytes, and similar to irisin, executed potent anti-obesity activities in an obese mouse model. Overall, our studies provided new mechanistic insights into GLP-1’s anti-obesity actions in which GLP-1 induces the secretion of FNDC5 derivatives from its responsive organs that then mediate its anti-obesity activities.

## Introduction

Obesity is a rising epidemic that puts people at higher risk for serious diseases such as diabetes, heart disease, and cancer (Engin, 2017). The US Food and Drug Administration (FDA) has approved multiple prescription medications to treat adults with body mass index (BMI) > 30 or >27 for those with weight-related comorbidity. These anti-obesity medications mainly reduce nutrient absorption or food intake. The latest approved anti-obesity therapeutic is liraglutide that is a glucagon-like peptide-1 (GLP-1) analog, or named GLP-1 receptor agonist (GLP-1RA). In healthy individuals, GLP-1 mediates the incretin effect, is essential for postprandial regulation of glucose, and is a major factor in regulating insulin secretion following oral glucose consumption. GLP-1 exerts its functions through binding to its cognate membrane-associated GLP-1R, whose gene was originally cloned from pancreatic β cells and later neurons (Campbell and Drucker, 2013). Ligand-bound GLP-1R initiates a signaling cascade to produce cyclic adenosine monophosphate (cAMP), which then activates different pathways in many tissues (e.g., pancreas and brain) to regulate glycemia, lipid metabolism, gut motility, and appetite (Rowlands et al., 2018). The beneficial properties of GLP-1 have led to the development of six FDA-approved GLP-1RAs (e.g., Rybelsus and liraglutide) for the treatment of type 2 diabetes (Hedrington and Davis, 2019). Importantly, tens of clinical trials have observed average weight reductions of 3–5 kg after taking GLP-1RAs over 20 weeks (Halawi et al., 2017; Christensen et al., 2019), resulting in the clinical use of a higher dose of liraglutide for weight management (3.0 mg vs. 1.2 or 1.8 mg for diabetes treatment). However, GLP-1RAs may cause dose-dependent side effects such as nausea (39% of patients), vomiting, and diarrhea (Gallwitz, 2015; Ladenheim, 2015; Nuffer and Trujillo, 2015; Suliman et al., 2019), correlating with poor patient compliance and weight regaining. Advanced mechanistic understanding of their anti-obesity actions can potentially facilitate their improved clinical use and the development of safer and more effective therapeutics for obesity control.

The molecular basis of anti-obesity actions of GLP-1 has been elucidated to a certain degree. GLP-1 can regulate apoptosis and pre-adipocyte proliferation, both of which are directly relevant to weight loss (Cantini et al., 2015). Furthermore, GLP-1 alters *de novo* lipogenesis and increases the expression of uncoupling protein 1 (UCP-1) and peroxisome proliferator-activated receptor gamma coactivator 1-α (PGC1α) in white adipose tissue (WAT) and brown adipose tissue (BAT) (Xu et al., 2016; Zhu et al., 2016). Interestingly, these actions are not related to nutrient intake but lead to increased energy expenditure. However, the expression of the GLP-1R gene in adipose tissue is conflicting in different studies, while its protein has not been unambitiously detected (Campbell and Drucker, 2013; Drucker, 2018). If GLP-1R is not present in adipose tissue, one potential scenario for GLP-1 to execute its anti-obesity actions is that its responsive organs release anti-obesity endocrine signaling molecules that act on adipose tissue. Supportively, the Nogueiras group recently revealed that liraglutide stimulates BAT thermogenesis and adipocyte browning in mice by activating AMPK in the brain hypothalamic ventromedial nucleus, irrelevant of food intake (Beiroa et al., 2014). On the other hand, anti-obesity signaling molecules released from the brain after liraglutide treatment remain unstudied.

Irisin is a recently identified hormone (Bostrom et al., 2012). Upon exercise or cold exposure, irisin is proteolytically released from the transmembrane protein fibronectin type III domain containing 5 (FNDC5) in multiple tissues, including skeletal muscle, fat, pancreas, and brain (Bostrom et al., 2012; Wrann et al., 2013). Two well-studied functions of irisin include the mediation of WAT browning and the increase of thermogenesis and lipid metabolism in WAT (Bostrom et al., 2012). Our team further uncovered the signaling of irisin’s browning activity that involves the activation of p38 mitogen-activated protein kinase (p38 MAPK) and ERK MAPK, leading to the overexpression of UCP-1 (Zhang et al., 2014). In addition, the lipolysis activity of irisin may be mediated by the cAMP-protein kinase A (PKA)/perilipin/hormone-sensitive lipase (HSL, encoded by LIPE gene) pathway (Xiong et al., 2015). Besides adipose tissue, irisin has multifaceted actions on other organs, e.g., promoting β cells survival and proliferation and stimulating insulin biosynthesis and secretion in the pancreas (Liu et al., 2017; Natalicchio et al., 2017). However, despite its significant health promise, irisin has been the subject of debate on its detection with commercially available antibodies and the existence of a full-length hormone in humans since its discovery (Albrecht et al., 2015; Albrecht et al., 2020). The latter is mainly related to the use of a non-canonical start codon in FNDC5 and the presence of diverse FNDC5 transcripts (Raschke et al., 2013; Albrecht et al., 2020). Nonetheless, native irisin has been detected in human plasma samples using western blotting with a commercial antibody and by quantitative mass spectroscopy (Jedrychowski et al., 2015), while the αV integrin receptors were recently identified to mediate irisin’s effects on bone and fat (Kim et al., 2018), addressing some concerns on its existence and physiological roles.

We realized that the anti-obesity effects of GLP-1 and GLP-1RAs notably overlap with those of FNDC5/irisin. Both GLP-1 and irisin inhibit adipocyte differentiation (Lee et al., 2015; Zhang et al., 2016), while stimulating the browning and lipolysis of WAT (Bostrom et al., 2012; Lockie et al., 2012; Beiroa et al., 2014). Furthermore, tissues that unambiguously express the GLP-1R gene and protein (e.g., brain and pancreas) also express the FNDC5 gene and protein (Wrann et al., 2013; Kim et al., 2017; Li Q. et al., 2019). More importantly, one recent clinical study involving 54 obese patients with type 2 diabetes revealed that the FDA-approved GLP-1RA exenatide significantly increases the serum level of irisin (Liu et al., 2016). However, the molecular link, if existing, between GLP-1 and FNDC5 for executing anti-obesity actions remains unknown. Herein, we report that FNDC5 derivatives can mediate GLP-1’s lipolysis effects. We observed that GLP-1 induces the overexpression of the FNDC5 gene and protein in human pancreatic β cells and the pancreas and brain of mice. Furthermore, GLP-1 induced the expression of lipolysis genes on β cells, which was dependent on the GLP-1 stimulated overexpression of the FNDC5 gene. Importantly, we found that β cells produce irisin and a novel secretable isoform of FNDC5 (sFNDC5), whose mRNA is further detected in multiple rat tissues. Similar to irisin, sFNDC5 possessed lipolysis action on β cells and adipocytes and executed anti-obesity effects in the mouse model. Our findings suggest that GLP-1 can induce the expression of FNDC5, leading to the production of its derivatives that likely execute the lipolysis action of GLP-1. These results provide fresh mechanistic insights into the overlapping anti-obesity actions of GLP-1 and FNDC5.

## Materials and Methods

### Cell Culture

The human pancreatic β-cell (βLox5), 3T3-L1, and HEK293 cells were maintained in Dulbecco’s modified Eagle’s medium (DMEM) supplemented with 10% fetal bovine serum (FBS) and 1% penicillin/streptomycin. INS-1 cells were cultured in RPMI-1640 medium containing 11.1 mM glucose supplemented with 10% fetal bovine serum (FBS), 100 U/ml penicillin, 100 μg/ml streptomycin, 10 mM HEPES, 2 mM L-Glutamine, 1 mM sodium pyruvate, and 50 μM β-mercaptoethanol. All cells were incubated at 37°C in a humidified atmosphere containing 5% CO_2_. 3T3-L1 cells were differentiated into mature adipocytes as previously described (Li H. et al., 2019).

### Reagents and Antibodies

GLP-1, SB203580 and KG-501 were purchased from Sigma-Aldrich. Liraglutide was purchased from Novo Nordisk (Tianjin, China). LY294002 and U0126 were purchased from Cell Signaling Technology. H89 was from Adipogen Life Science. Anti-LC3A/B, anti-pERK1/2, anti-ERK1/2, anti-pAKT, and anti-AKT antibodies were purchased from Cell Signaling Technology. Anti-FNDC5 (ab174833) and anti-ATGL were from Abcam, while anti-UCP-1 and anti-β-actin antibodies were purchased from Sigma-Aldrich. HRP-conjugated secondary antibodies for western blotting were purchased from Cell Signaling Technology. Irisin-competitive ELISA kit (AG-45A-0046YEK-KI01) was from Adipogen Life Science.

### Plasmid Construction and Luciferase Assays

For the *FNDC5* promoter-luciferase reporter constructs, the human *FNDC5* proximal promoters of 1279, 859, or 684 bp were cloned into pGL4.10 (luc2) (Promega). HEK293 cells were transfected with 1.0 μg of the construct with *FNDC5*-luciferase reporter gene and cotransfected with 0.1–1.0 μg of the constructs carrying transcription factors (as indicated). Cell lysates were harvested and measured 24 h post-transfection using the Dual Luciferase reporter kit (Promega). All luciferase assays were done in triplicate.

### CRISPR/Cas9-Mediated FNDC5 Knockout

The four pairs of sgRNAs specifically targeting exon 3 of the human *FNDC5* gene were designed using the online Target Finder program (http://tools.genome-engineering.org) and the sequences are listed in Supplementary Table S1. To construct CRISPR/Cas9 plasmid vectors targeting *FNDC5*, the four sgRNA DNA fragments were individually ligated into the lentiCRISPRv2puro vector (#98290; Addgene) to produce infectious viral particles. βlox5 cells were transfected with the lentiviruses for 48 h and then treated with 2 μg/ml puromycin for 4–6 days. Drug-resistant cells were detached with trypsin and separated into single cells. Cells were seeded onto 96-well plates at a density of 1 cell/well. To identify the presence of indels in the *FNDC5* gene, the genomic DNA was extracted for PCR amplification of the target site with the primers list in Supplementary Table S1.

### Expression and Purification of Rat sFNDC5 Protein From Pichia Pastoris

We followed our established protocols to produce r-sFNDC5 (Zhang et al., 2014).

### Preparation of Cells Expressing GFP-LC3

βLox5 cells were transiently transfected with the GFP conjugated LC3 plasmid (#22405; Addgene) using Lipofectamine 2000 (ThermoFisher, Invitrogen). The cells were incubated for 24 h and treated with GLP-1 or r-sFNDC5 for 24 h. The images were obtained by fluorescence microscopy.

### Western Blot Analysis

Total proteins were extracted from snap-frozen adipose tissue (∼50 mg) or cell lysates, and 20–50 μg of proteins were loaded into precast SDS-PAGE gels (4–20%) and transferred onto PVDF membranes. The membranes were blocked with 5% skim milk in Tris-buffered saline, pH 7.4, containing 0.1% Tween 20, at room temperature for 1 h, and were incubated with specific primary antibodies at 4°C overnight. The antibodies were diluted to their appropriate ratio according to manual instructions. Membranes were washed and a secondary antibody conjugated to HRP was used for western blotting. Experiments were performed in triplicate and a representative blot was shown.

### Animal Studies

Male Sprague-Dawley rats (200 g) were used to detect the FNDC5 gene expression in various tissues (*n* = 3). Six-week-old male C57BL/6 mice were purchased from the Model Animal Research Center of Nanjing University. Animal studies were performed according to the National Institutes of Health Guide for the Care and Use of Laboratory Animals and approved by the Ethics Committee of the Second Hospital of Shandong University. From the age of 8 weeks onwards, the mice were divided into two groups and fed with a normal chow diet or high-fat diet (HFD) (Trophic diet, China, #TP23520) respectively for 10 weeks. HFD mice were randomly divided into four groups according to the case number and treated with recombinant sFNDC5 (r-sFNDC5) (0.5 μg/g), recombinant irisin (r-irisin) (0.5 μg/g), liraglutide (1 μg/g), or saline by intraperitoneal (i.p.) injection once daily from 9 to 10 a.m. for 14 days. Chow diet-fed mice received i.p. injections of saline at the same volume. The body weight and tolerance tests of the mice were measured after 14 days. In the GTT, the mice were intraperitoneally injected with 2 g glucose/kg after an overnight fast. In the ITT, the mice were injected with insulin (0.75 U/kg) after a 6-h fasting. The tail vein blood glucose was subsequently measured at 0, 15, 30, 60, and 120 min for both GTT and ITT. Liver, epididymal (visceral), and subcutaneous adipose tissues were flash-frozen in liquid nitrogen and stored at −80°C for RNA and protein extraction. Tissues for hematoxylin and eosin (H&E) staining, immunohistochemistry (IHC), and Oil Red O staining were immediately fixed in 4% paraformaldehyde and then embedded, sliced, and stained. Peripheral blood was collected to determine lipid metabolic profiles. To detect GLP1-induced FNDC5 expression in mouse pancreas and brain tissue, C57BL/6 mice were fed with chow diet and randomly divided into two groups, and treatment with either liraglutide or saline as control were given once daily by i.p. injection for 8 days. Mice were sacrificed at the end of the day, pancreas and brain tissues were collected for RNA and protein extraction. Serum irisin levels were measured by using ELISA kits.

### Statistical Analysis

All data are presented as the means ± SEM. The statistical significance was analyzed by using GraphPad Prism 7.0 software, and a comparison between two groups was performed using one-way ANOVA analysis followed by an unpaired Student’s t-test. *p* < 0.05 was considered to be statistically significant.

## Results

### GLP-1 Induces FNDC5 Expression in Human Pancreatic β Cells

**FIGURE 1:**
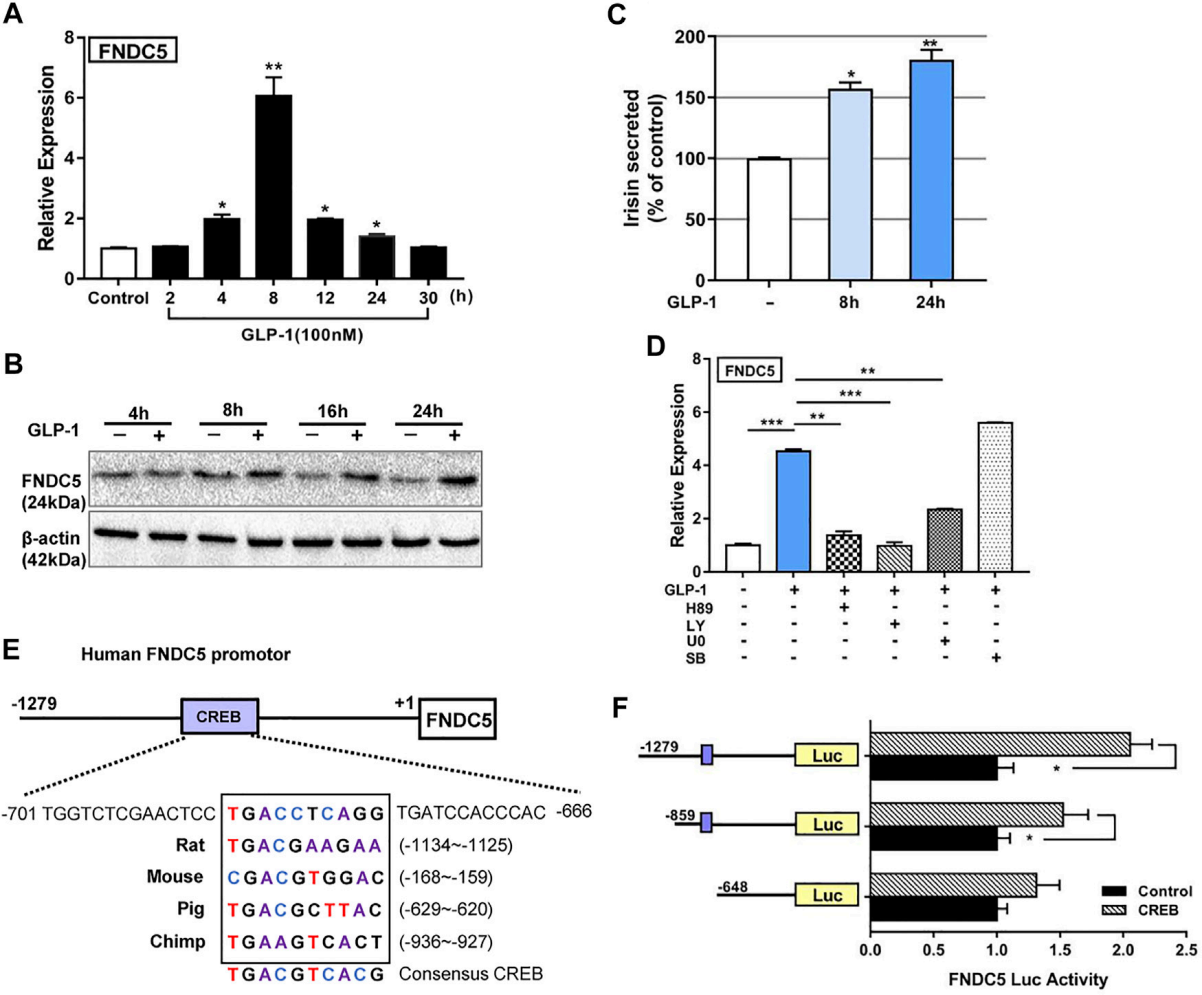
GLP-1 upregulates FNDC5 gene expression in human pancreatic β-cells and mouse pancreas and brain tissue. The expression levels of FNDC5 mRNA **(A)** and protein **(B)** in GLP-1-treated (100 nM) βLox5 cells were analyzed at indicated time points. **(C)** Secreted irisin in the culture medium of GLP-1-treated (100 nM) βLox5 cells was measured by using ELISA. The level in untreated controls was set as 100% for normalization. **(D)** The expression level of *FNDC5* mRNA in βLox5 cells. Cells were treated with GLP-1 (100 nM) and selected inhibitors for 8 h before RT-qPCR analysis. **(E)** A conserved CREB binding site in the *FNDC5* gene promoter from multiple mammalian species. The prediction was performed with the online Genomatix Suite Collection. The box-enclosed regions highlight the predicted core CREB binding motif, and the consensus sequence is shown at the bottom. **(F)** CREB regulates the expression of luciferase (Luc) under the control of the *FNDC5* promoter. HEK293 cells were transfected with a *CREB* expression plasmid (0.5 μg) and a plasmid with luciferase under the control of three different lengths of the *FNDC5* promoter (1 μg). Values are mean ± SEM. *p < 0.05, **p < 0.01 and ***p < 0.001.

A primary target of GLP-1 is pancreatic islet β cells, while the pancreas is also known to express the FNDC5 gene (Kim et al., 2017). To assess the potential functional relationship of GLP-1 and FNDC5 in β cells, we treated the human pancreatic β cell line βLox5 with GLP-1 (100 nM). Following treatment, the transcription level of FNDC5 was significantly increased from 4 to 24 h and peaked at 8 h with an approximately 5-fold increase (Figure 1A). GLP-1 treatment also upregulated FNDC5 protein expression at 8, 16, and 24 h (Figure 1B), leading to the increased level of secreted FNDC5 derivatives in the cell culture media as measured by an enzyme-linked immunosorbent assay (ELISA) with an irisin-specific antibody (Figure 1C). The GLP-1-induced expression of the FNDC5 gene and protein was also observed in the rat β cell line INS-1 (Supplementary Figures S1A,B), which expressed a higher level of insulin gene upon GLP-1 treatment (Supplementary Figure S1C).

Next, we sought to characterize signaling pathways by which GLP-1 induces the expression of FNDC5 in β cells. The ERK, PKA, and AKT signaling cascades play major roles in exerting the biological effects of GLP-1 in β cells (Rowlands et al., 2018), and can thus potentially be involved in activating FNDC5 expression. Accordingly, we treated βLox5 cells with GLP-1 (100 nM) alone or with a pathway-specific inhibitor H89 (PKA), LY294002 (AKT), U0126 (ERK), or SB203580 (p38). Except for the p38 inhibitor, the PKA, AKT, and ERK inhibitors all significantly suppressed the GLP-1 induced activation of three key lipolysis genes, adipose triglyceride lipase (patatin-like phospholipase domain containing protein 2, encoded by Pnpla2; hereafter referred to as ATGL), HSL, and hepatic lipase C (LIPC) (Supplementary Figure S2), suggesting their mediation of the lipolysis actions of GLP-1 in β cells. Importantly, these inhibitors also suppressed the GLP-1 induced expression of FNDC5 to the basal level (Figure 1D), while SB203589 showed no inhibition. These results indicated that the ERK, PKA, and AKT pathways all are required to mediate the GLP-1-induced expression of FNDC5 in β cells.

We sought to obtain an additional understanding of the transcriptional regulation of FNDC5 by GLP-1. Our bioinformatics analysis identified a putative binding site element (TGACCTCAGG) of cAMP response element-binding protein (CREB) at −687 to −678 bp from the transcriptional start site of the human FNDC5 gene, which is highly conserved in rats, mice, pigs, and chimpanzees (Figure 1E). CREB is an important transcription factor known to mediate multiple actions of GLP-1 in pancreatic islets (Shin et al., 2014; Chen et al., 2017). To examine if this element regulates the transcription of FNDC5, we fused the 1279-bp promoter region of the human FNDC5 gene 5′-terminal to the firefly luciferase gene. The luciferase signal increased with the dose of the CREB gene in cotransfected HEK293 cells (Figure 1F and Supplementary Figure S3A), and the signal increase depended on the presence of the predicted CREB binding site in the FNDC5 promoter (Figure 1F). These results demonstrated that CREB can potentially regulate the GLP-1-induced expression of FNDC5 in β cells. As PGC1-α was shown to upregulate the expression of FNDC5 in myocytes through the formation of a PGC1-α/CREB complex (Yang et al., 2018), we cotransfected HEK293 cells further with PGC1-α. Interestingly, we observed minimal regulatory effects of expressed PGC1-α directly on the FNDC5 promotor alone or with CREB (Supplementary Figure S3B), leading to no significant activation of luciferase expression. In alignment with this result, we found that four other known β cell-specific transcription factors PDX1, NGN3, MAF, and HNF1α may not bind to the FNDC5 promoter to activate the gene expression as the luciferase signal was not increased in HEK293 cells when cotransfected with their genes (Supplementary Figure S3C). To further verify the regulation role of CREB, we treated INS-1 cells with GLP-1 (100 nM) alone or with the CREB specific inhibitor KG-501 (10 µM). As expected, KG-501 treatment reduced the GLP-1 induced transcription of FNDC5 to the basal level (Supplementary Figure S3D). Collectively, the above results indicated that CREB interacts with its binding element in the FNDC5 promotor to activate its expression in GLP-1 treated β cells.

## GLP1 Induces FNDC5 Expression in Mouse Pancreas and Brain Tissue

**FIGURE 2:**
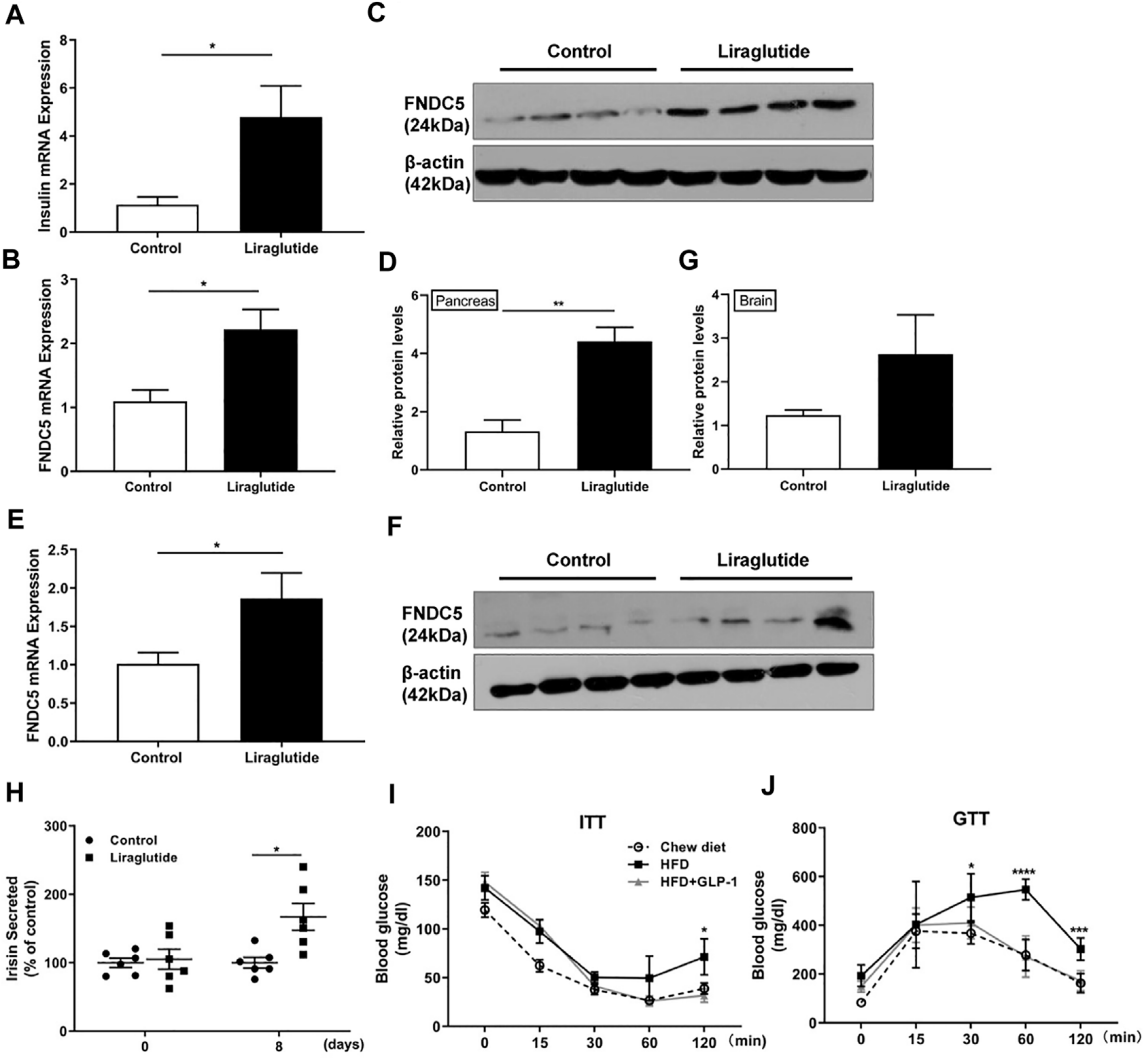
GLP-1 upregulates FNDC5 gene expression in mouse pancreas and brain tissue. C57BL/6 mice (*n* = 4–6) were fed with chow diet and treated with either liraglutide (1 μg/g) or saline as control once daily by i.p. injection. Pancreas and brain tissues were collected from mice after 8 days of treatment. **(A)** The expression of the insulin gene was measured in the pancreas. The expression levels of FNDC5 mRNA and protein in the pancreas **(B**–**D)** and brain tissues **(E**–**G)** were analyzed by RT-qPCR and western blotting. The expression level in control was set as 1 for normalization. β-Actin expression was used as a control in western blotting analysis. **(H)** The serum levels of secreted irisin in two mice groups (*n* = 6) were measured by ELISA. The level in the control was set as 100% for normalization. **(I**,**J)** HFD-induced obese C57BL/6 mice were treated with liraglutide for 14 days and ITT and GTT were performed (*n* = 6–8). Values are mean ± SEM. *p < 0.05, **p < 0.01, ***p < 0.001 and ****p < 0.0001.

To further characterize the functional relationship between GLP-1 and FNDC5, we treated 6-week-old C57BL/6 mice (*n* = 4) with one clinically used GLP-1RA liraglutide (1 μg/g) once daily by i.p. injection for 8 days. As expected, liraglutide markedly increased the expression of insulin mRNA in the mouse pancreas at the end of the treatment (Figure 2A). Importantly, it further upregulated the expression of FNDC5 mRNA and protein in the pancreas (Figures 2B–D), agreeing with the results from the human and rat β cell lines (Figure 1). In addition to the pancreas, accumulating evidence suggests the brain as another important GLP-1 responsive organ that can mediate GLP-1’s anti-obesity actions (Beiroa et al., 2014; Secher et al., 2014; Sisley et al., 2014; Smith et al., 2019). Indeed, we also observed the increased expression of FNDC5 mRNA and its protein in mouse brain tissue after 8-day liraglutide treatment (Figures 2E–G). Furthermore, the liraglutide treatment led to a significantly higher serum level of secreted FNDC5 derivative than the control as quantitated by the irisin ELISA assay (Figure 2H and Supplementary Figure S4), aligning well with the 50% increase of serum irisin in patients with T2D after the treatment of GLP-1RA exenatide for 12 weeks (Liu et al., 2016). We further verified the biological activity of liragultide (1 μg/g) with obese C57BL/6J mice (*n* = 6) that were fed with high fat diet (HFD) for 10 weeks. After daily injection for 14 days, liraglutide ameliorated the impaired glucose tolerance and insulin sensitivity of these obese mice (Figures 2I,J). Taken together, these results revealed that GLP-1 induces the expression of FNDC5 in both mouse pancreas and brain tissue, which correlates with the higher level of FNDC5 derivatives in the circulation after liraglutide treatment.

## FNDC5 Mediated GLP-1 Induced Lipolysis Gene Expression in β Cells

**FIGURE 3:**
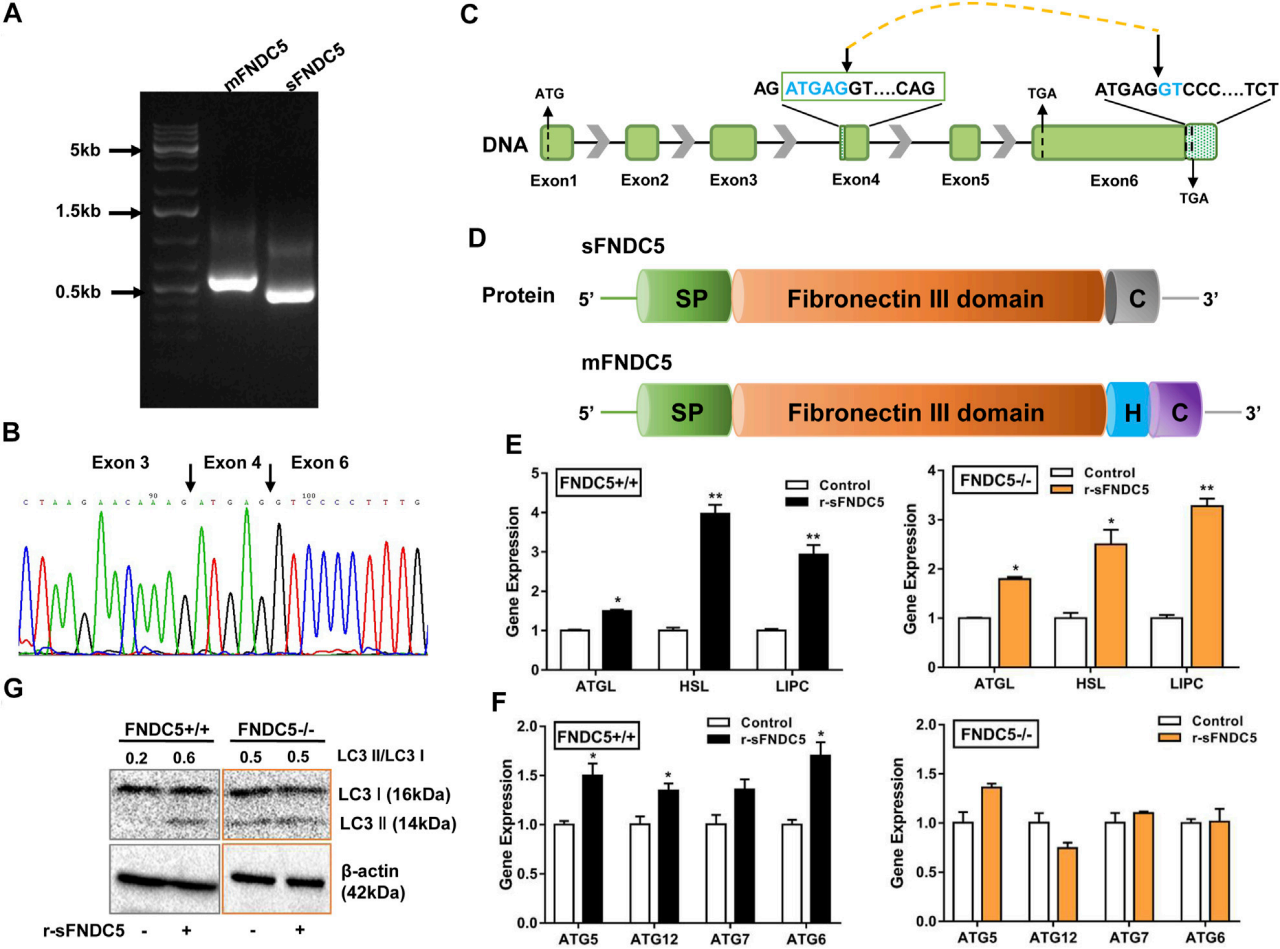
A secreted isoform of FNDC5 (sFNDC5) mediates GLP-1 induced lipolysis and autophagy of β cells. **(A)** INS-1 cells expressed a long (*mFNDC5*) and a short *FNDC5* (*sFNDC5*) transcript in agarose analysis. **(B)** Sequencing chromatograph of the novel secreted FNDC5 isoform. **(C**,**D)** Schematic representation of the gene and protein structure of sFNDC5 and mFNDC5. SP, signal peptide; H, hydrophobic transmembrane domain; C, C-terminal domain. **(E,F)** r-sFNDC5 (50 nM) induced the expression of lipolysis genes but not autophagy-related genes in FNDC5 KO βLox5 cells. Wild-type cells were used as the control. Cells were treated with r-sFNDC5 (50 nM) for 16 h (*n* = 3). **(G)** r-sFNDC5 (50 nM) induced the expression of LC3 I/II in wild type but not FNDC5 KO βLox5 cells. Cells were treated with r-sFNDC5 (50 nM) for 24 h (*n* = 3). β-Actin expression was used as a control in western blotting analysis. Each experiment was repeated three times. Values are mean ± SEM. *p < 0.05 and **p < 0.01.

Glucose stimulated insulin secretion (GSIS) occurs in pancreatic β cells when the ATP/ADP ratio is increased to cause a rise of intracellular Ca2+ concentration (Prentki and Matschinsky, 1987) and cAMP is formed to trigger insulin granule exocytosis through the activated PKA (Seino et al., 2009). Lipid molecules are also involved in GSIS. Previous research and our present studies indicate that GLP-1 triggers triglyceride hydrolysis (lipolysis) in β cells *via* activating the expression of HSL and ATGL (Supplementary Figure S2) (Peyot et al., 2004; Sorhede Winzell and Ahren, 2004; Tang et al., 2013). Interestingly, it is also well known that irisin stimulates the lipolysis of adipocytes through upregulating the expression of HSL, ATGL and fatty acid-binding protein 4 (FABP4) (Huh et al., 2014; Xiong et al., 2015; Rodriguez et al., 2017), but its lipolysis function in β cells remains unstudied. To interrogate the potential relationship between GLP-1 and FNDC5 in regulating the lipid metabolism of β cells, we inactivated the FNDC5 gene in βLox5 cells by introducing 1- or 2-bp insertions or deletions in its exon 3 with a CRISPR/Cas9 mediated gene editing approach (Supplementary Figures S5A,B). Western blotting analysis with an irisin-specific antibody revealed no expression of FNDC5 protein (about 24 kD) in the FNDC5 knockout (KO) cells (Figure 3A). Furthermore, GLP-1 treatment of the KO β cells for 24 h no longer induced the secretion of FNDC5 derivatives into the culture medium (Figure 3B), different with the wild type cells (Figure 1C). These results validated the successful knockout of FNDC5 in βLox5 cells. Next, we treated the wild-type and FNDC5 KO βLox5 cells with GLP-1 (100 nM) to examine the activation of the lipolysis program. Compared with the wild type, the mutant cells showed no activated expression of three key lipolysis genes, ATGL, HSL, and LIPC after GLP-1 treatment (Figure 3C), while GLP-1 still upregulated the transcription of the truncated FNDC5 mRNA. These results suggested that GLP-1 first induces the expression of FNDC5 and the expressed intact FNDC5 or its secreted derivatives subsequently stimulate the lipolysis genes in β cells. This newly discovered functional relationship between GLP-1 and FNDC5 advances the mechanistic understanding of GLP-1 induced lipolysis in β cells. Interestingly, GLP-1 treatment also failed to activate the expression of autophagy-related genes (ATG5, 6, 7, and 12) and proteins (LC3 I and II) in the FNDC5 KO βLox5 cells (Figures 3D,E). Autophagy is a highly conserved intracellular principal catabolic pathway that is essential to β cell survival and maintenance of normal functions (Levine and Klionsky, 2004; Arden, 2018). Nutrient deprivation/starvation are well known to activate the autophagy, which delivers intracellular proteins and organelles sequestered in double-membrane vesicles to lysosomes for degradation and use as an energy source (44). Moreover, lipid metabolism is also regulated by autophagy in β cells (Singh et al., 2009). Indeed, LC3 I and II were gradually overexpressed in both wild type and FNDC5 KO βLox5 cells after starvation for 2 days (Figure 3F). These results suggested that GLP-1 and FNDC5-initiated autophagy was inhibited in FNDC5 KO β-cells, while other pathways of autophagy remain unaffected. Collectively, our studies revealed that GLP-1-induced expression of FNDC5 is critical to the lipolysis and autophagy actions of GLP-1 in β cells.

## A New Secreted FNDC5 Derivative Activates GLP-1-Induced Lipolysis in β Cells

**FIGURE 4:**
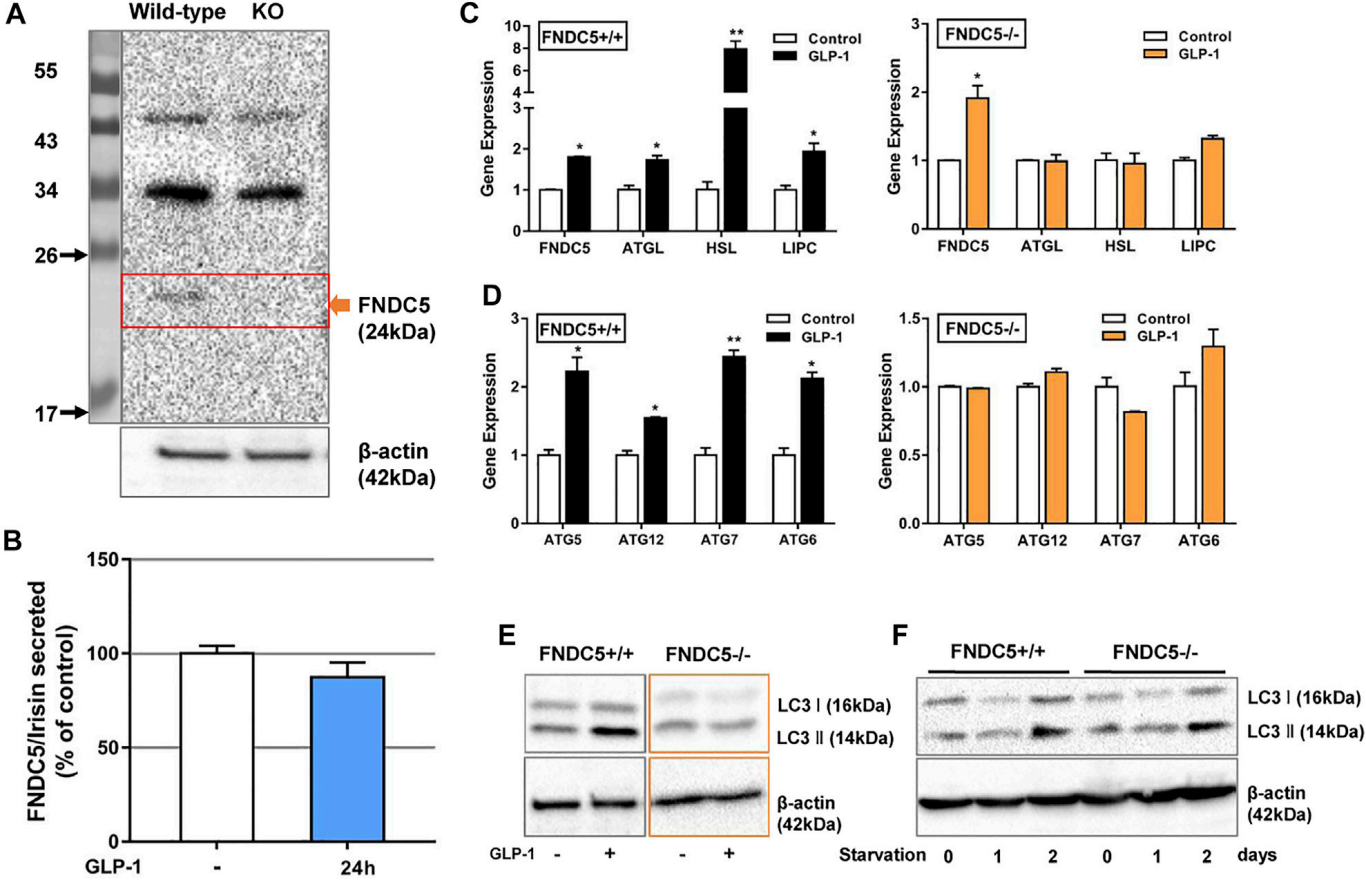
FNDC5 mediates the GLP-1 induced lipolysis genes expression and autophagy of β cells. **(A)** FNDC5 protein was not expressed in its KO βLox5 cells. β-Actin expression was used as a control in western blotting analysis. **(B)** GLP-1 (100 nM) failed to increase the secretion of FNDC5/irisin in FNDC5 KO βLox5 cells (*n* = 3). GLP-1 failed to activate the expression of lipolysis genes **(C)** and autophagy-related genes **(D)**. Wild type or FNDC5 KO βLox5 cells were treated with GLP-1 (100 nM) for 16 h before RT-qPCR analysis (*n* = 3). **(E)** The expression of LC3 I/II was not enhanced by GLP-1 in FNDC5 KO βLox5 cells. Wild type or FNDC5 KO βLox5 cells were treated with GLP-1 (100 nM) for 24 h before western blotting analysis (*n* = 3). **(F)** βLox5 cells were cultured in FBS free medium for indicated time and anti-LC3 I/II antibodies were used to probe cell lysates following SDS-PAGE. β-Actin expression was used as a control. Values are mean ± SEM. *p < 0.05 and **p < 0.01.

As the FNDC5 gene is known to produce diverse transcripts (Raschke et al., 2013; Albrecht et al., 2020), we probed the possibility of the generation of novel secreted FNDC5 derivatives that carry the irisin component but are not produced from membrane-bound FNDC5 (mFNDC5). Indeed, we identified a new FNDC5 isoform transcript from rat INS-1 cell lines by RT-qPCR analysis (Figure 4A), and the primers are listed in Supplementary Table S1. The novel isoform is derived from a repeat sequence (ATGAGGT) in both exon 4 and exon 6 following a mechanism for the regulation of pre-mRNA alternative splicing (Figures 4B,C) (Huh and Hynes, 1994; Hui et al., 2005). The new isoform with 167 amino acids shares the almost identical fibronectin III domain as irisin, lacks the transmembrane domain (exon 5), and carries a distinctly different C-terminus from mFNDC5 (Figure 4D and Supplementary Figure S6). To avoid confusion, we named this isoform as sFNDC5. We also detected both mFNDC5 and sFNDC5 transcripts in all tested organs of Sprague Dawley rats (*n* = 3) (Supplementary Figure S7). These two transcripts showed a similar level in the liver, skeleton muscle, and heart, while we observed 3–6-fold higher expression of sFNDC5 in the spleen, lung, and adipose tissue. Collectively, these results supported the presence of a readily secretable FNDC5 isoform in β cells and rats.

To characterize the potential biological function of sFNDC5, we prepared its recombinant protein (r-sFNDC5) using a Pichia pastoris expression system and the protein was purified from the clear medium using a Ni-NTA chromatography column (Zhang et al., 2014). SDS-PAGE analysis showed purified r-sFNDC5 as five bands of 18–27 kDa, and PNGase F treatment led to a single band at around 18 kDa, suggesting serial N-glycosylation modifications (Supplementary Figure S8). Remarkably, r-sFNDC5 (50 nM) significantly enhanced the expression of ATGL, HSL, and LIPC in both wild-type and FNDC5 KO βLox5 cells (Figure 4E). This result indicated that sFNDC5 may be produced upon GLP-1 treatment and then mediate its lipolysis actions in β cells (Figures 3C, 4E). On the other hand, similar to GLP-1, r-sFNDC5 induced the expression of autophagy-related genes ATG5, ATG6, ATG7, and ATG12 and LC3 I and LC3 II proteins in wild-type but not FNDC5 KO βLox5 cells (Figures 4F,G). To further investigate the role of r-sFNDC5 in GLP-1-induced autophagy, we transfected wild-type and FNDC5 KO βLox5 cells with the GFP-LC3 construct. Compared with untreated cells, both GLP-1 (100 nM) and r-sFNDC5 (50 nM) induced a higher level of green fluorescence puncta in the wild type but not FNDC5 KO βLox5 cells (Supplementary Figure S9).

## Secreted FNDC5 Derivatives but Not GLP-1 Induce the Browning and Lipolysis of Differentiated Adipocytes

**FIGURE 5:**
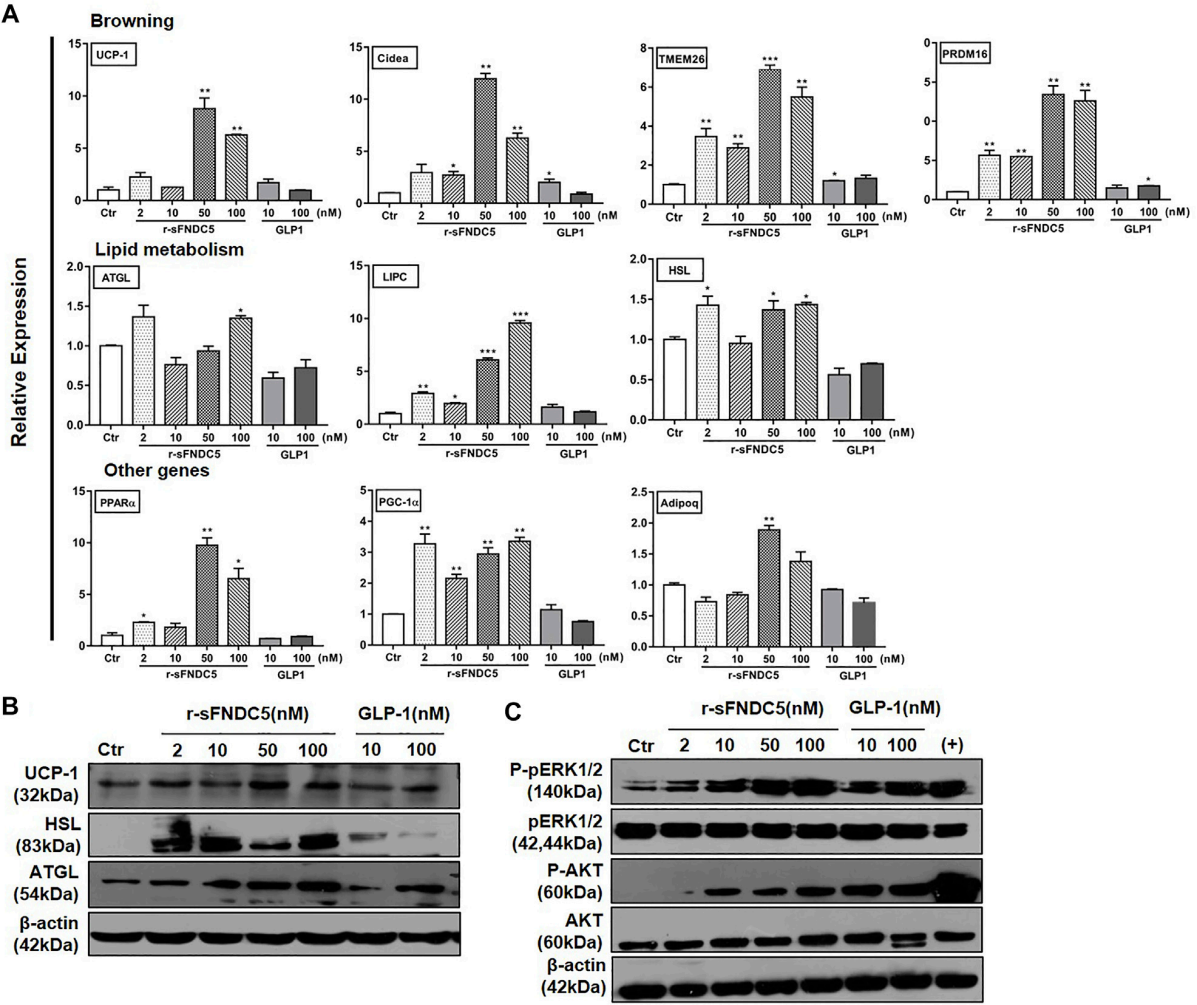
The anti-obesity activities of GLP-1 and sFNDC5 in mature 3T3-L1 adipocytes. **(A)** Upregulated expression of selected biomarker genes of browning, lipolysis, and other programs in fully differentiated, mature 3T3-L1 adipocytes by r-sFNDC5 and GLP-1. The cells were treated with r-sFNDC5 and GLP-1 at indicated concentrations for 24 h (*n* = 3). Untreated cells served as the control for normalizing the signals. **(B)** Upregulated expression of UCP-1, HSL, and ATGL in mature 3T3-L1 adipocytes by r-sFNDC5 and GLP-1 at indicated concentrations. Cells were treated with r-sFNDC5 and GLP-1 for 24 h (*n* = 3). Untreated cells served as control. β-Actin expression was used as a control in western blotting analysis. **(C)** Activation of ERK1/2 and AKT in mature 3T3-L1 adipocytes by r-sFNDC5 and GLP-1 at indicated concentrations. Cells were treated with r-sFNDC5 and GLP-1 for 24 h (*n* = 3). Untreated cells served as control. β-Actin expression was used as a control in western blotting analysis. Values are mean ± SEM. *p < 0.05, **p < 0.01 and ***p < 0.001.

The anti-obesity actions of GLP-1/GLP-1RAs are expected to restore energy balance by reducing energy intake and/or increasing energy consumption. The latter is particularly significant to reduce enlarged adipose tissue in obese patients. Indeed, GLP-1 has been shown to regulate adipogenesis, lipolysis, and BAT thermogenesis (Xu et al., 2016), although the presence and expression of GLP-1R in adipocytes remain conflicting in different studies (Campbell and Drucker, 2013; Pastel et al., 2016; Chen et al., 2017). On the other hand, the current work revealed that the GLP-1RA induces the expression of FNDC5 in the mouse pancreas and brain and increases the serum level of secreted FNDC5 derivatives (Figure 2). To test the extent to which secreted FNDC5 derivatives mediate GLP-1’s anti-obesity actions on adipose tissue, we treated mature 3T3-L1 adipocytes with serial concentrations of r-sFNDC5 and GLP-1. r-sFNDC5 strongly induced the expression of browning-related genes UCP-1, PRDM16, Cidea, and TMEM26 in a dose-dependent manner with the maximal activity at 50 nM, while GLP-1 (10 and 100 nM) showed only a weak browning activity (Figure 5A). Furthermore, r-sFNDC5 but not GLP-1 induced the expression of lipolysis-related genes (ATGL, HSL, and LIPC) and proteins (UCP-1, HSL, and ATGL) in mature 3T3-L1 adipocytes (Figures 5A,B). We further observed that r-sFNDC5 stimulated the expression of key genes involved in fatty acid β-oxidation (PPARα), mitochondrial biogenesis (PGC1α), and one anti-inflammatory and insulin-sensitizing adipokine (Adipoq) (Figure 5A) and activated the ERK and AKT signaling pathways (Figure 5C). These results suggested that r-sFNDC5 possesses anti-obesity activity, similar to irisin (Xiong et al., 2015), and more importantly, these secreted FNDC5 derivatives may mediate the lipolysis effects of GLP-1 on adipose tissue.

## sFNDC5 Executes Anti-Obesity Activities in an Obesity Mouse Model

**FIGURE 6:**
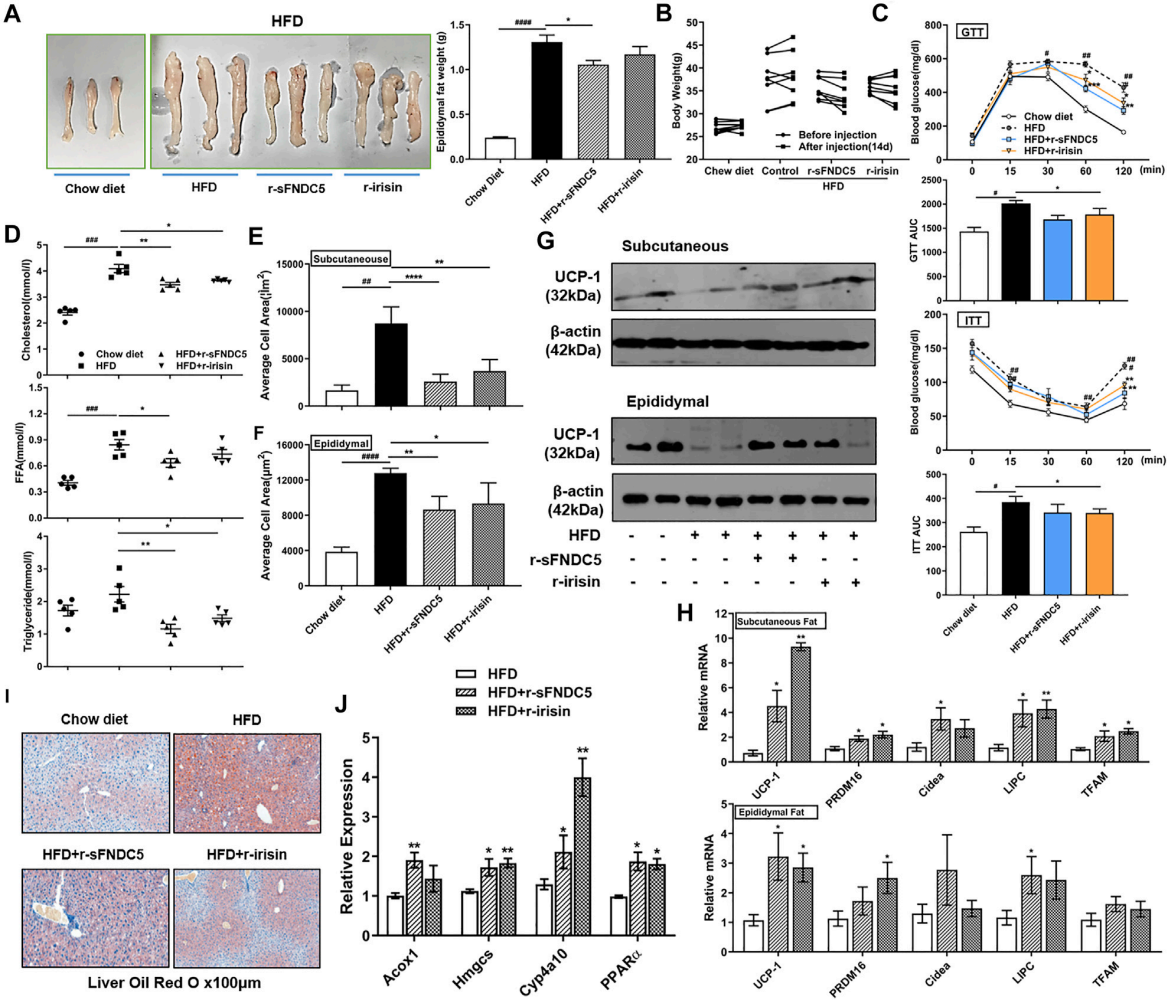
r-sFNDC5 attenuated diet-induced obesity, glucose metabolism/insulin sensitivity, and dyslipidemia. HFD induced obese C57BL/6 mice were i.p. injected with r-sFNDC5, r-irisin (0.5 μg/g/day), or saline daily for 14 days, and C57BL/6 mice fed with chow diet were injected with saline as a negative control (*n* = 4–9/group). **(A)** Morphology (left) and weight (right) of epididymal fat from different mice groups. **(B)** Body weights, **(C)** glucose tolerance test, (GTT) and insulin tolerance test (ITT) of mice before and after injection of saline, r-sFNDC5, or r-irisin for 14 days. **(D)** Serum levels of cholesterol, triglyceride, and free fatty acid (FFA) levels in different mice groups. Mice were fasted overnight before the measurement. **(E**,**F)** Quantification of mean adipocyte size in subcutaneous and epididymal fat in different mice groups. **(G)** r-sFNDC5 and r-irisin upregulated the expression of UCP1 in subcutaneous and epididymal fat from different mice groups. β-Actin expression was used as a control in western blotting analysis. **(H)** Expression of selected biomarker genes of browning and lipolysis in subcutaneous and epididymal fat from different mice groups. **(I)** r-sFNDC5 and r-irisin reduced the lipid accumulation in liver sections of obese mice, and **(J)** expression of fatty acid oxidation-related gene expression in livers from different mice groups. Values are mean ± SEM. #*p* < 0.05, ##*p* < 0.01 ###*p* < 0.001 and ####*p* < 0.0001 vs. chow diet mice; *p < 0.05, **p < 0.01 and ***p < 0.001 vs. HFD mice.

We further characterized the anti-obesity activity of sFNDC5 using a high fat diet (HFD)-induced obesity mouse model. C57BL/6 mice after 10-weeks HFD challenge (*n* = 4–9) were injected daily with r-sFNDC5 (0.5 μg/g/day), recombinant irisin (r-irisin, 0.5 μg/g/day) that we previously prepared (Zhang et al., 2014), or saline. The treatment lasted for 14 days. Compared with the saline control, both r-sFNDC5 and r-irisin reduced the weight of mouse epididymal fat and the body weight of HFD mice (0.28–4.59 g) (Figures 6A,B). R-sFNDC5 showed more significant effects than r-irisin. Furthermore, both r-sFNDC5 and r-irisin significantly improved glucose tolerance in obese mice as demonstrated in the glucose tolerance test (GTT) and significantly reduced levels of fasting insulin in the insulin tolerance test (ITT) (Figure 6C). These two secreted FNDC5 derivatives further decreased the serum levels of cholesterol, triglyceride, and free fatty acid (FFA) (Figure 6D). It is reasonable to speculate that sFNDC5 not only induces lipolysis in adipocytes but also promotes glycerol and lipid consumption in other organs. Hematoxylin and eosin (H&E) staining of subcutaneous and epididymal adipose tissues revealed that r-sFNDC5 and r-irisin reduced the enlarged adipose tissues in obese mice (Figures 6E,F and Supplementary Figures S10A,B). UCP-1 is a key biomarker of BAT, and both r-sFNDC5 and r-irisin induced the expression of its mRNA and protein in subcutaneous and epididymal adipose tissues (Figures 6G,H and Supplementary Figures S10A,B). In addition, r-sFNDC5 and r-irisin upregulated the transcription of other metabolism-related genes including PRDM16, Cidea, LIPC, and TFAM (Figure 6H). Furthermore, we found that both r-sFNDC5 and r-irisin promote fat oxidation in the liver, as indicated by decreased hepatic lipid accumulation and increased expression of fatty acid β-oxidation genes (Figures 6I,J). Collectively, these results highlight that similar to irisin, sFNDC5 exerts anti-obesity effects in diet-induced obese mice by reducing fat mass and enhancing the lipolytic and oxidative capacity of WAT.

## Discussion

It is well known that oral glucose ingestion and exercise induce the secretion of GLP-1 into the circulation (Ueda et al., 2009a; Ueda et al., 2009b; Martins et al., 2015; Holliday and Blannin, 2017; Hira et al., 2020). The primary roles of this peptide hormone include the enhancement of glucose-stimulated insulin secretion and lipid metabolism after directly binding to its receptor on the surface of pancreatic β cells. The mechanisms of its regulation of glucose homeostasis have been thoroughly described (Drucker, 2018). In contrast, how GLP-1 mediates lipid metabolism remains incompletely understood.

**FIGURE 7:**
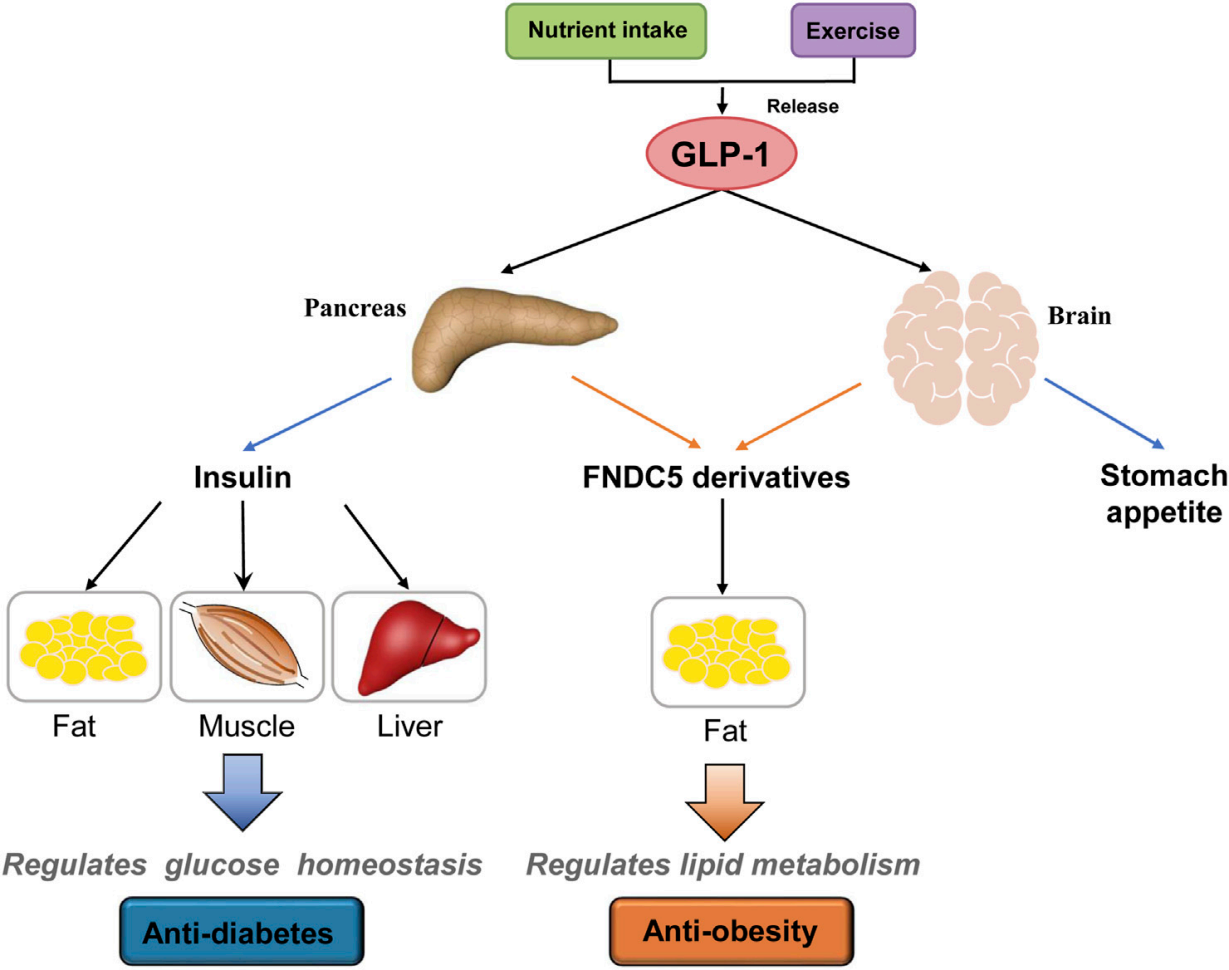
A proposed model of GLP-1’s anti-diabetes and anti-obesity actions. GLP-1 is produced after food intake and/or exercise and acts on the pancreas, brain, and other responsive organs. In the pancreas, GLP-1 induces the release of insulin that then regulates glucose homeostasis through its actions on fat, muscle, and liver. On the other hand, GLP-1 induces the secretion of FNDC5 derivatives (e.g., sFNDC5 and irisin) from the pancreas and brain that regulate lipid metabolism in adipose tissue. GLP-1 also suppresses appetite through its action on the brain. Other potential organs involved in GLP-1 actions are not shown.

The GLP-1R has been clearly observed in brain tissue and the pancreas (Smith et al., 2019). On the other hand, RNA-seq, *in situ* hybridization, or GLP-1R antisera analysis showed no to low expression of GLP-1R in hepatocytes, skeletal myocytes, and adipocytes (Campbell and Drucker, 2013; Panjwani et al., 2013). More recently, one study suggested that GLP-1’s function in adipocytes is mediated by GLP-1R and an additional, unknown receptor (El Bekay et al., 2016). In this work, we observed no direct lipolysis effects of GLP-1 on mature 3T3-L1 adipocytes. In contrast, sFNDC5 and irisin, possessed potent anti-obesity effects on adipocytes and HFD-induced obese mice (Figures 5, 6). sFNDC5 and irisin can be produced upon GLP-1 treatment of pancreas, brain tissue, and possibly other organs and then circulated to adipose tissues. These results suggest one possibility in which GLP-1 regulates lipid metabolism indirectly through its induced production of downstream molecules or hormones (Figure 7), analogous to the GLP-1 induced release of insulin from the pancreas to lower the blood glucose level. Interestingly, the Sisley group generated mice with glutamatergic neuronal deletion of GLP-1R and observed that GLP-1R agonists successfully execute glucose-lowering effects but fail to induce weight loss (Sisley et al., 2014), suggesting that the brain may control the anti-obesity actions of GLP-1. The brain is one major tissue expressing FNDC5 (Kim et al., 2017), and the current work clearly indicated the upregulated expression of FNDC5 in the brain of mice after daily injection of liraglutide (1 μg/g) for 8 days (Figures 2D,E). Furthermore, we observed a higher serum level of secreted FNDC5 derivatives after liraglutide treatment (Figure 2F). The results from previous and current studies likely imply that GLP-1 can induce the secretion of FNDC5 from the brain to execute its lipolysis actions in adipose tissue (Figure 7). On the other hand, this work does not exclude other possibilities, for example other GLP-1 responsive organs might also release FNDC5 derivatives or other hormones with anti-obesity activities.

The current work discovered a novel FNDC5 isoform, sFNDC5, that is expressed along with mFNDC5 in β cells and multiple rat organs. The *N*-terminus of sFNDC5 is identical irisin but this new hormone carries a new, 31-amino acid peptide tail at its *C*-terminus. The biological consequence of the tail remains unclear but sFNDC5 demonstrated similar activities to irisin in the obese mouse model (Figure 6). There are diverse FNDC5 transcripts reported to date (Raschke et al., 2013; Albrecht et al., 2020), but all irisin-containing transcripts carry the transmembrane domain. However, the protease for the release of irisin from the transmembrane protein remains unknown since the discovery of irisin in 2012 (Bostrom et al., 2012). sFNDC5 is the first readily secretable FNDC5 derivative. Further studies are needed to characterize the circulating level, distribution, and biological functions of sFNDC5 in humans, supporting its basic and translational applications. Furthermore, recombinant sFNDC5 prepared in the yeast expression system demonstrated potent biological activities but it remains unclear about the effects of glycosylation modifications on its biological functions in animals and humans.

In conclusion, this work shows that GLP-1 exerts its lipolysis effects on pancreatic β-cells by inducing the expression and production of secreted FNDC5 derivatives. GLP-1 further induced the expression of FNDC5 in brain tissues. Importantly, we reported the discovery of sFNDC5, a secretable FNDC5 isoform, that possesses potent lipolysis actions on pancreatic β cells, adipocytes, and the diet-induced obese mouse model. Lipid metabolism regulated by secreted FNDC5 derivatives carries important implications regarding the mechanism of GLP-1RAs as anti-obesity drugs. Future studies are required to obtain additional insights into the functional and mechanistic relationships between GLP-1 and FNDC5 and to investigate the therapeutic potential of sFNDC5.

Guarantor: Dr. Shiwu Li is the guarantor of this work and has full access to all the data in the study and takes responsibility for the integrity of the data and the accuracy of the data analysis.

## Data Availability

The original contributions presented in the study are included in the article/Supplementary Material, further inquiries can be directed to the corresponding authors.
