# Supplementary material for: GLP-1 Induces the Expression of FNDC5 Derivatives That Execute Lipolytic Actions
Source: Front Cell Dev Biol. 2021 Nov 11;9:777026. doi: 10.3389/fcell.2021.777026 (PMC8636013; doi:10.3389/fcell.2021.777026)
Supplement: Supplementary file 2 [file Table1.DOCX]

**Supplementary Materials**

**Table S1. The list of primers used in this work**

| **(1) single-guide RNA (sgRNA)** | | |
| --- | --- | --- |
| **sgRNA1** | caccGCTCTTCAAGACCCCGCGTG | aaacCACGCGGGGTCTTGAAGAGC |
| **sgRNA2** | caccGCCATCTTCTCAGCCTCACG | aaacCGTGAGGCTGAGAAGATGGC |
| **sgRNA3** | caccGGTCCCAGAGGGCACATGAG | aaacCTCATGTGCCCTCTGGGACC |
| **sgRNA4** | caccGGAGTACATAGTCCACGTGC | aaacGCACGTGGACTATGTACTCC |
| **(2) Primers used for indel detection of target sites in exon 3** | | |
| **FNDC5-F:** CCGAGTGTTGACAGCAGTGACC | | |
| **FNDC5-R:** TATGCTCAGCGTCTTGTCAGG | | |
| **(3) Primers used for sFNDC5/mFNDC5 detection** | | |
| **FNDC5-F1:** ATGCCCCCAGGGCCGTGCGCCTG (5’) | | |
| **FNDC5-R1:** TCATATCTTGCTTCGGAGGAGACCC (3’-end of mFNDC5) | | |
| **FNDC5-R2:** TCAGGCCTTGGCAGAGCCCTCTGC (3’-end of sFNDC5) | | |
| **(4)** **Primers used for gene expression analysis by RT-qPCR** | | |
| **Human Gene** | **Forward Primer** | **Reverse Primer** |
| *β-actin* | GACCTCTATGCCAACACAGT | AGTACTTGCGCTCAGGAGGA |
| *FNDC5* | ATCTCCATTCAGGGCCAGAG | GGTTCCTCCCCATCTCTTTC |
| *ATGL* | CTGCTGATAGCCATGAGCATG | CTTAAGCTCATAGAGTGGCAGG |
| *HSL* | GGTCACAGATTCTGAGTCAG | GTCATTGTGCGCAGGTCCATG |
| *LIPC* | CTGGATCTGGCAGATGGTGG | ATGGCTTCGAGAGAGTTGCAC |
| *ATG5* | AACTGAAAGGGAAGCAGAACCA | CCATTTCAGTGGTGTGCCTTC |
| *ATG7* | CAGTTTGCCCCTTTTAGTAGTGC | CTTAATGTCCTTGGGAGCTTCA |
| *ATG6* | TCCATGCTCTGGCCAATAAGA | TGTCAGAGACTCCAGATATGAATGGT |
| *ATG12* | ATTGCTGCTGGAGGGGAAGG | GGTTCGTGTTCGCTCTACTGC |
| **Mouse Gene** | **Forward Primer** | **Reverse Primer** |
| *β-actin* | TGAGCTGCGTTTTACACCCT | GCCTTCACCGTTCCAGTTTT |
| *Acox1* | CCTGATTCAGCAAGGTAGGG | TCGCAGACCCTGAAGAAATC |
| *Hmgcs* | ATACCACCAACGCCTGTTATG | CAATGTCACCACAGACCACCA |
| *Cyp4a10* | AAGGGTCAAACACCTCTGGA | GATGGACGCTCTTTACCCAA |
| *PPARα* | CAGTGGGGAGAGAGGACAGA | AGTTCGGGAACAAGACGTT |
| *UCP-1* | AGTACCCAAGCGTACCAAGC | ATGATGACGTTCCAGGACCC |
| *TFAM* | ATTAGGAGGGTCTCGCTCCA | GGCCATGCAAGGCTTTTCC |
| *PRDM16* | CCCCACATTCCGCTGTGAT | CTCGCAATCCTTGCACTCA |
| *Cidea* | TGCTCTTCTGTATCGCCCAGT | GCCGTGTTAAGGAATCTGCTG |
| *TMEM26* | TTCCTGTTGCATTCCCTGGTC | GCCGGAGAAAGCCATTTGT |
| *ATGL* | GACAGCTCCACCAACATCCA | GCAAAGGGTTGGGTTGGTTC |
| *HSL* | GTGGCGAAAAGGCAAGATCA | TTCCCGAACACCTGCAAAGA |
| *LIPC* | CTTGCAACACAGTGACCTGC | CGGTCCTTGCGGATGTCATA |
| *PGC1α* | CCCTGCCATTGTTAAGACC | TGCTGCTGTTCCTGTTTTC |
| *Adipoq* | GCACTGGCAAGTTCTACTGCAA | GTAGGTGAAGAGAACGGCCTTGT |
| *Insulin* | GTGACCAGCTATAATCAGAGAC | CAGGTAGAGAGCCTCTACCAGG |
| **Rat Gene** | **Forward Primer** | **Reverse Primer** |
| *Insulin* | CCCTAAGTGACCAGCTACAATC | CAGGTACAGAGCCTCCACCAGG |

**
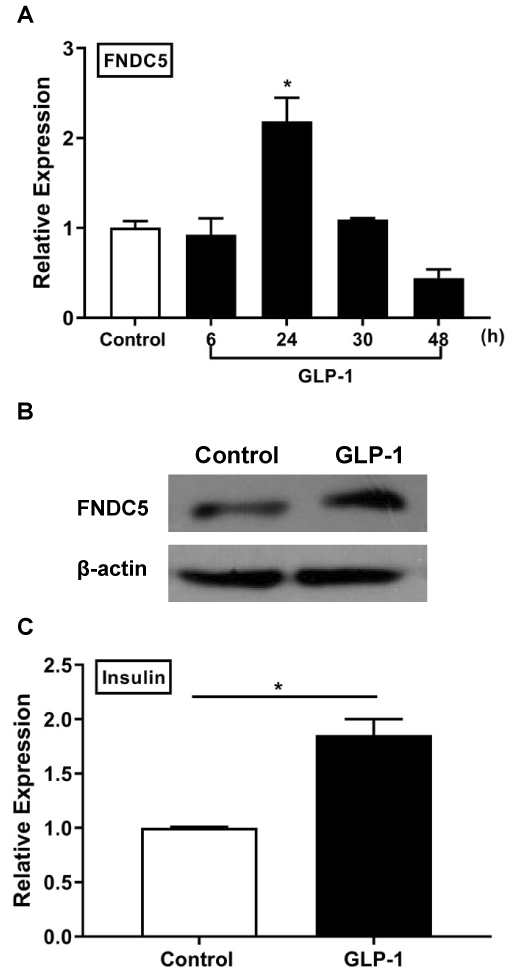
**

**Figure S1. GLP-1 upregulates *FNDC5* gene expression in rat INS-1 pancreatic β-cells.** The expression levels of FNDC5 mRNA **(A)** and protein **(B)** in GLP-1-treated (100 nM) INS-1 cells at indicated time points. (**C**) The expression of insulin gene in GLP-1-treated (100 nM) INS-1 cells. Cells were incubated with GLP-1 or saline for 24 h. Each experiment was repeated three times. Values are mean ± SEM. *P<0.05.

**

**

**Figure S2. Effects of cell signaling pathway inhibitors on the expression of GLP-1 induced lipolysis genes in βLox5 cells.** βLox5 cells were treated with GLP-1 (100 nM) alone or 10 nM indicated cell signaling pathway inhibitors for 16 h. Untreated cells served as the control. Each experiment was repeated three times. Values are mean ± SEM. *P<0.05 and **P<0.01.


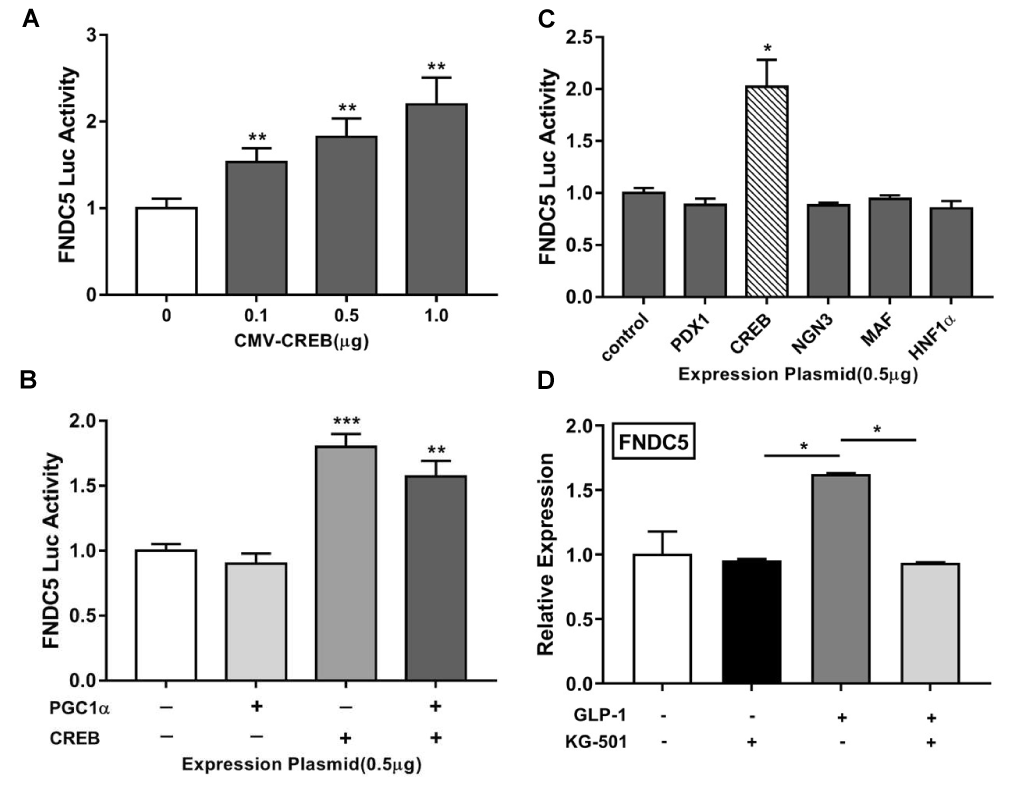


**Figure S3. CREB activates gene expression under the control of the *FNDC5* promoter. (A)** The luciferase signals in HEK293 cells transfected with the *FNDC5* promoter-luciferase reporter plasmid (1 μg) and serial concentrations of the *CREB* expression plasmid. **(B)** PGC1α does not activate the expression of luciferase. HEK293 cells were co-transfected with the reporter plasmid (1 μg), *PGC1α*, and/or *CREB* expression plasmids (0.5 µg). **(C)** Selected β cell-specific transcription factors fail to activate the expression of luciferase. HEK293 cells were co-transfected with the reporter plasmid (1 μg) and transcription factor expression plasmids (0.5 µg). Luciferase signal was measured 24 h post-transfection. (**D**) The CREB inhibitor KG-501 inhibits the upregulated expression of *FNDC5* by GLP-1 in INS-1 cells. Cells were treated with 100 nM GLP-1 with or without KG-501 (10 μM) for 24h before RT-qPCR analysis. Each experiment was repeated three times. Values are mean ± SEM. *P<0.05, **P<0.01 and ***P<0.001.





**Figure S4. Liraglutide increases the serum level of secreted FNDC5/irisin.** Mice were treated with different concentrations of vehicle or liraglutide for 8 days (n = 6). Secreted FNDC5/irisin in serum was measured using ELISA and expressed as a percentage to vehicle-treated controls. Values are mean ± SEM. *P<0.05


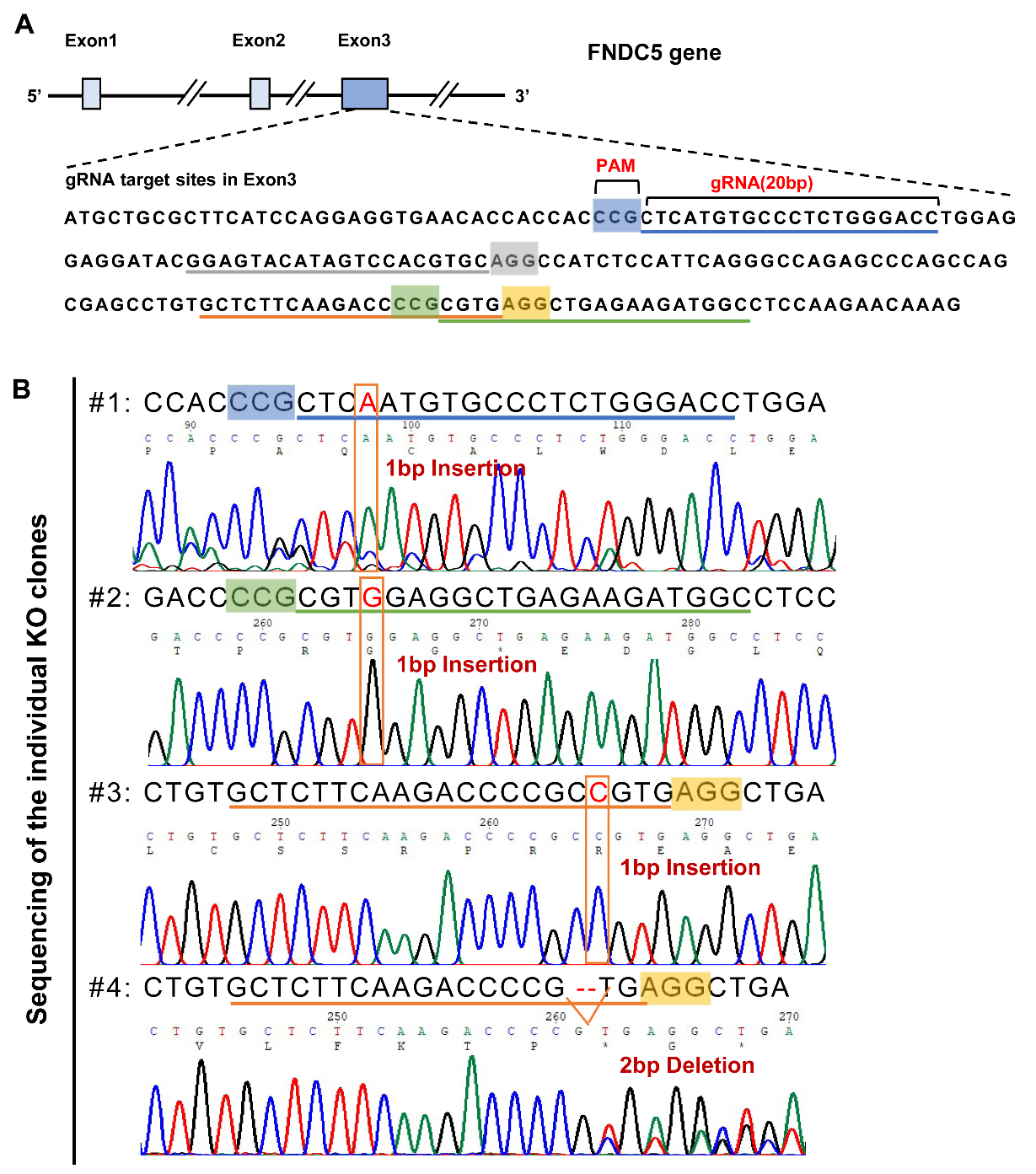


**Figure S5. CRISPR/Cas9-mediated *FNDC5* KO in β cells. (A)** Schematic depiction of sgRNA-guided Cas9 target site within exon 3 of the FNDC5 gene. Four sgRNA-targeting sequences are underlined with lines of different colors and their PAMs are shadowed in the same color. sgRNA, single-guide RNA; PAM, pattern adjacent motif. **(B)** DNA sequencing analysis of four clones revealed three nucleotide insertions and one nucleotide deletion in CRISPR/Cas9-edited human βlox5 clones. The black text, red text, and dash represented the original FNDC5 sequence, inserted nucleotide, and deleted nucleotides, respectively.


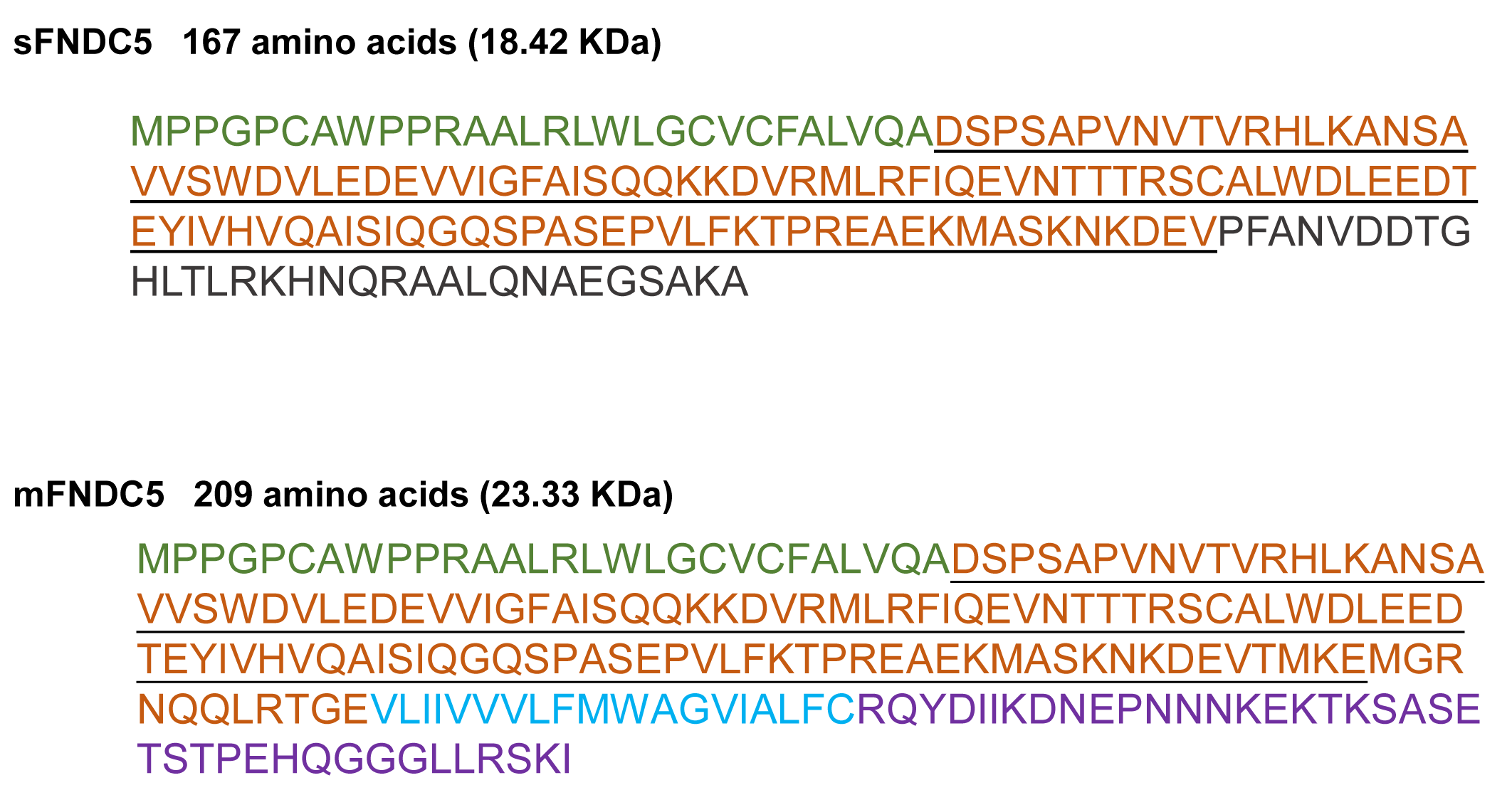


**Figure S6. The discovery of a secreted isoform of FNDC5 (sFNDC5) in the INS-1 cell line.** Amino acid sequence of secreted FNDC5 (sFNDC5, top) and membrane-bound FNDC5 (mFNDC5, bottom). FNDC5 amino acid sequences are colored according to corresponding domains shown in **Figure 4D**. The irisin sequence is underlined. Comparing with irisin derived from mFNDC5, sFNDC5 lacks its *C*-terminal TMKE but carries a new 31-amino acid tail.





**Figure S7. Expression of mFNDC5 and sFNDC5 in multiple rat tissues.** The expression of sFNDC5 (black bars) and mFNDC5 (white bars) mRNA transcripts was quantitated in indicated rat tissues using isoform specific primers by RT-qPCR (n=3). The relative expression level was normalized to mFNDC5. Values are mean ± SEM. *P<0.05 and **P<0.01.


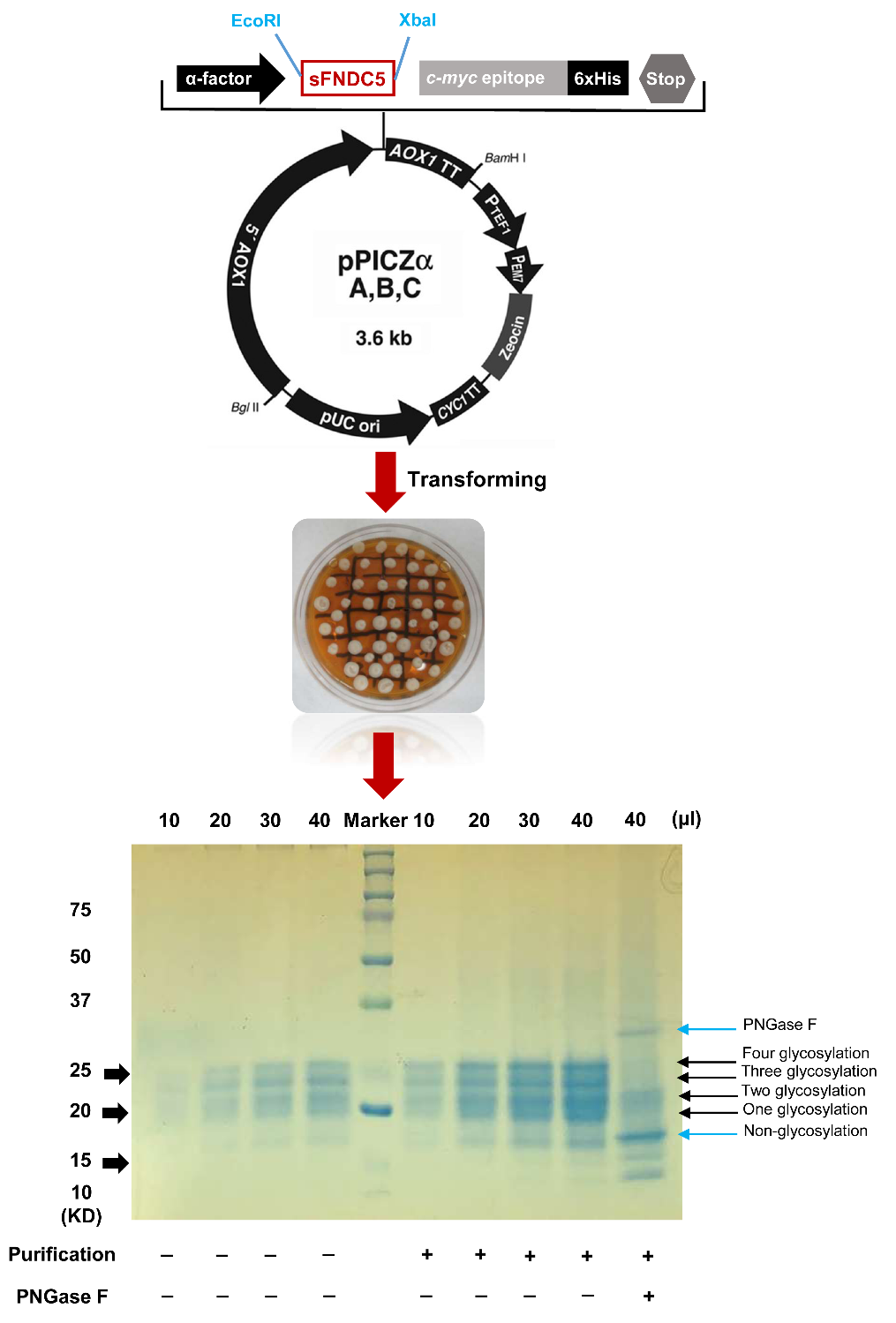


**Figure S8. Expression of r-sFNDC5.** The synthesized codon-optimized rat sFNDC5 cDNA was cloned into the EcoRI/XbaI sites of the pPICZαA plasmid. *Pichia pastoris* X-33 was transformed with a linearized pPICZαA-sFNDC5 plasmid. The yeast culturing and induction of protein expression were performed according to the manual instruction. The r-sFNDC5 in the supernatant was purified by Ni-NTA chromatography column. PNGase-F treatment of r-sFNDC5 followed the manual instruction.


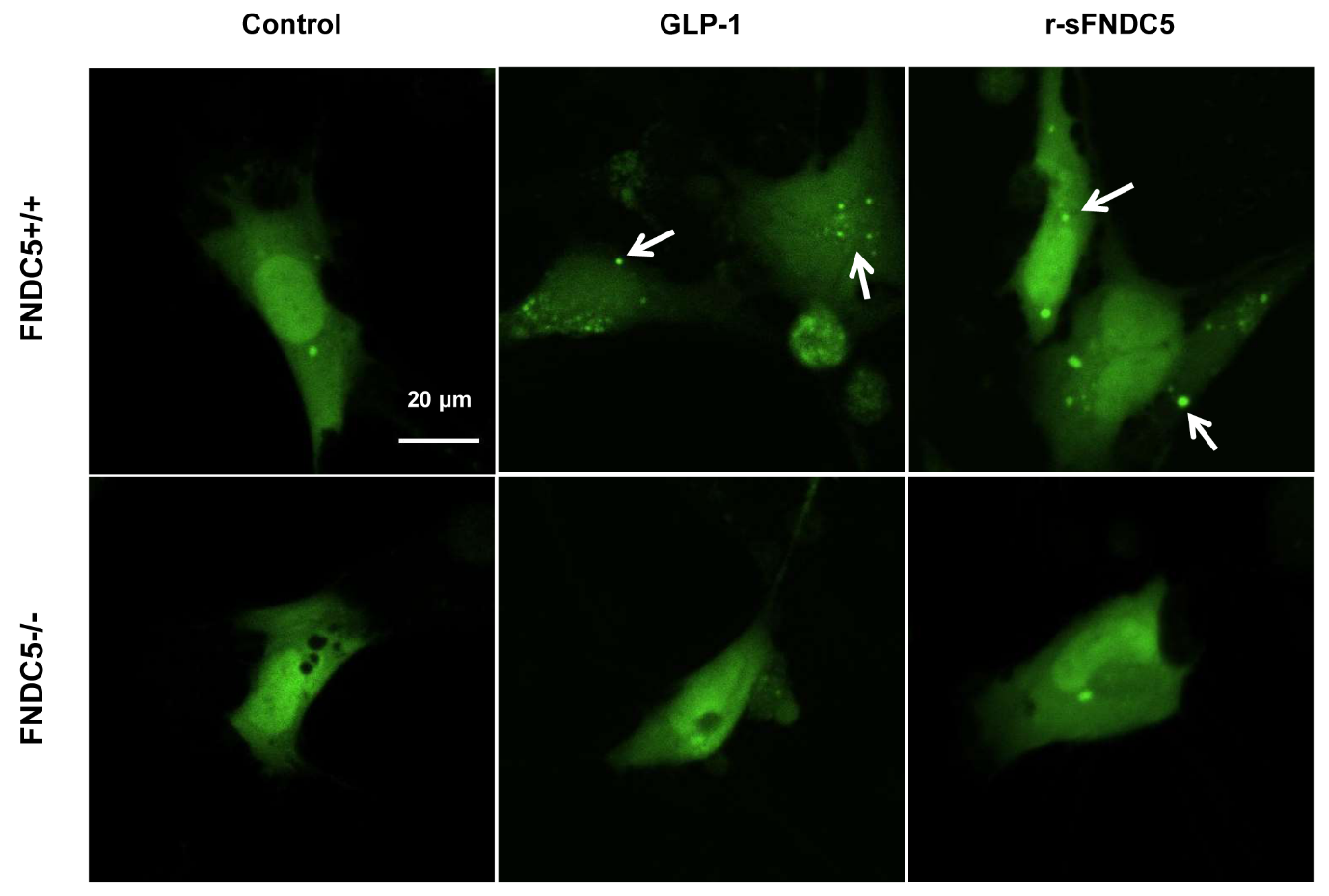


**Figure S9. The effect of GLP-1 and r-sFNDC5 on autophagy of β cells.** βLox5 cells were transfected with the GFP-LC3 expression plasmid and then treated with GLP-1 or r-sFNDC5 for 24 h. Cells were observed under fluorescence microscopy (magnification, x200). Scale bar, 20 μm. The cell autophagic puncta are shown by the bright green dots.


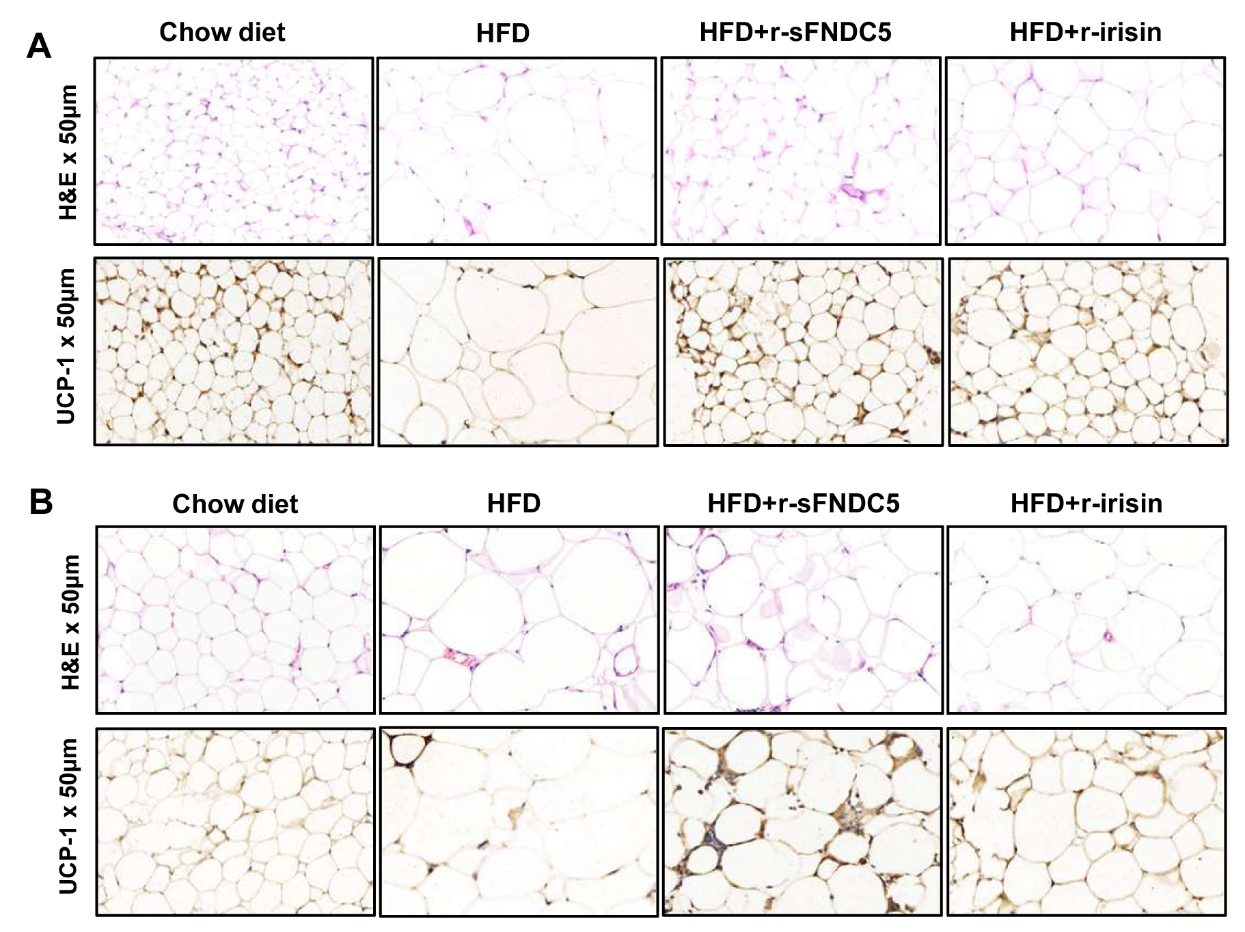


Figure S10. r-sFNDC5 and r-irisin treatment changed the body adiposity phenotype in HFD mice. H&E staining (upper panel) and IHC staining for UCP-1 (lower panel) in subcutaneous (A) and epididymal fat tissue (B).
