# Supplementary figures and images for: GLP-1 Induces the Expression of FNDC5 Derivatives That Execute Lipolytic Actions
Source: Front Cell Dev Biol. 2021 Nov 11;9:777026. doi: 10.3389/fcell.2021.777026 (PMC8636013; doi:10.3389/fcell.2021.777026)

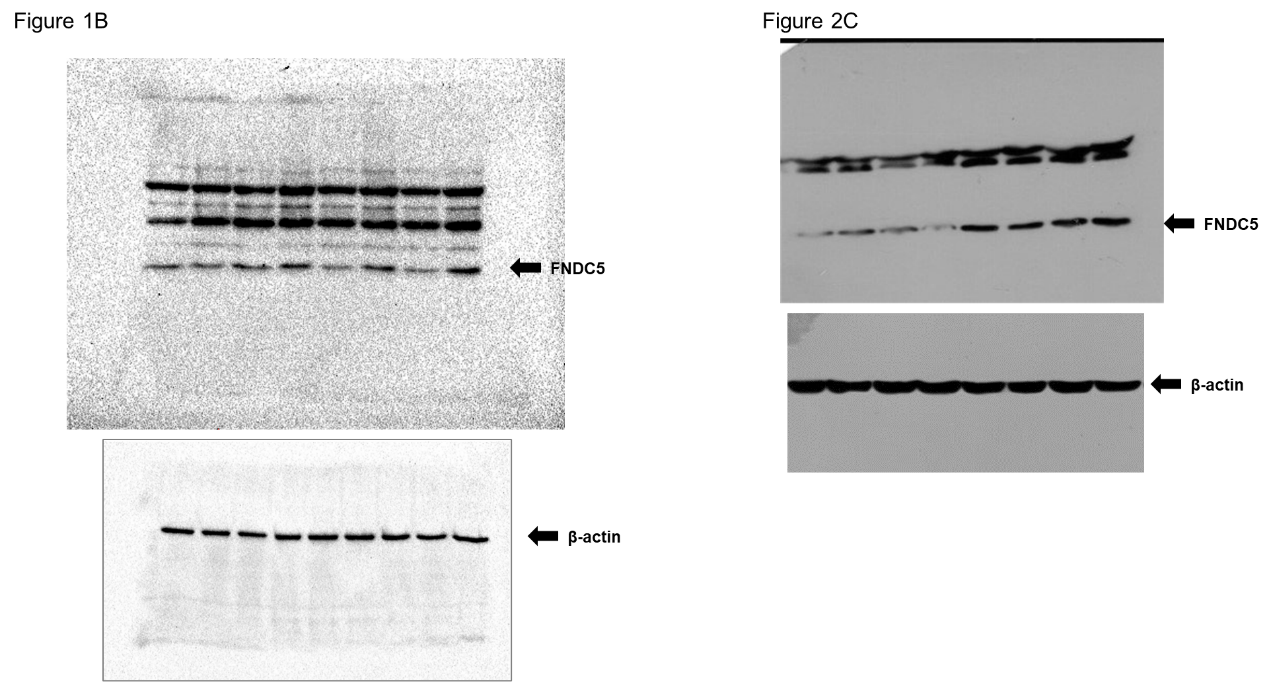


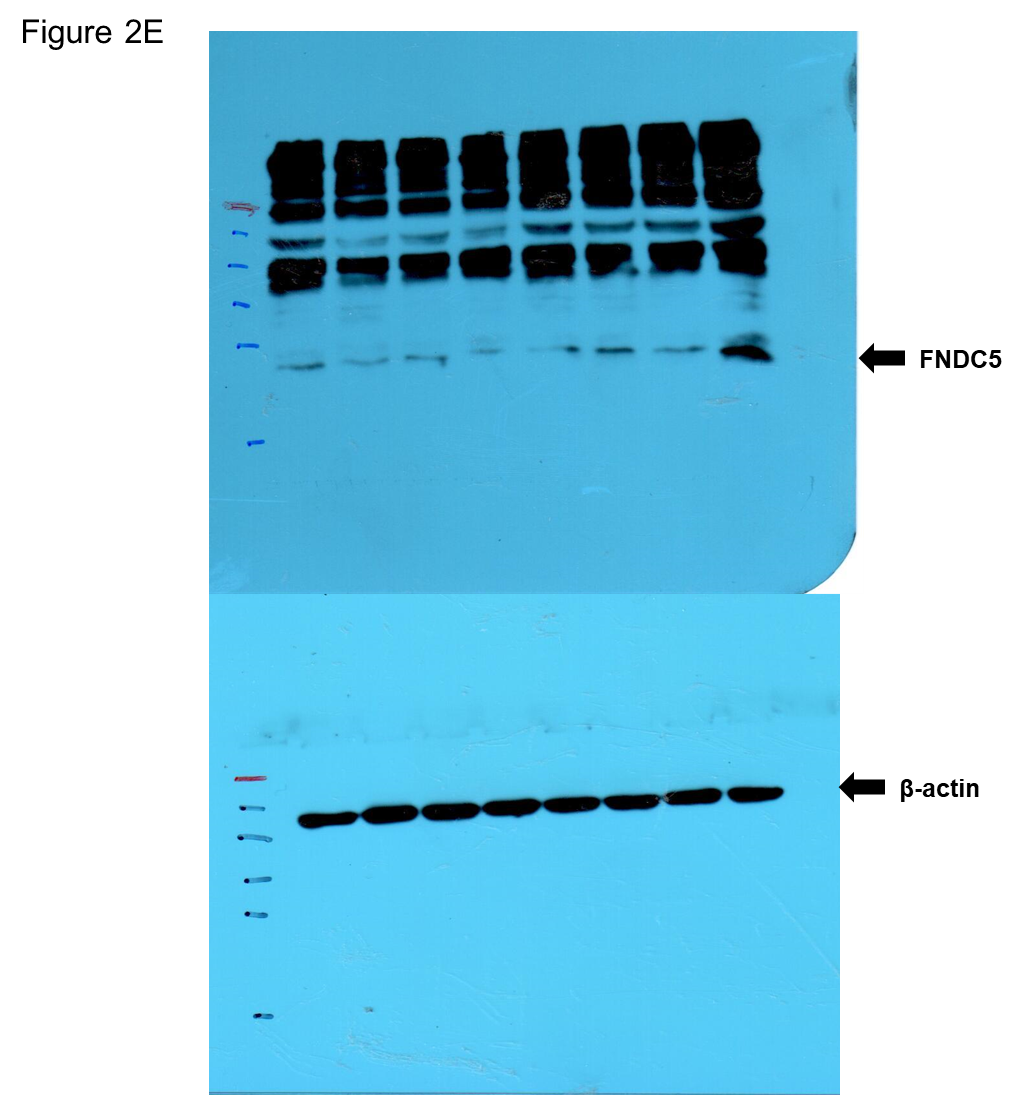


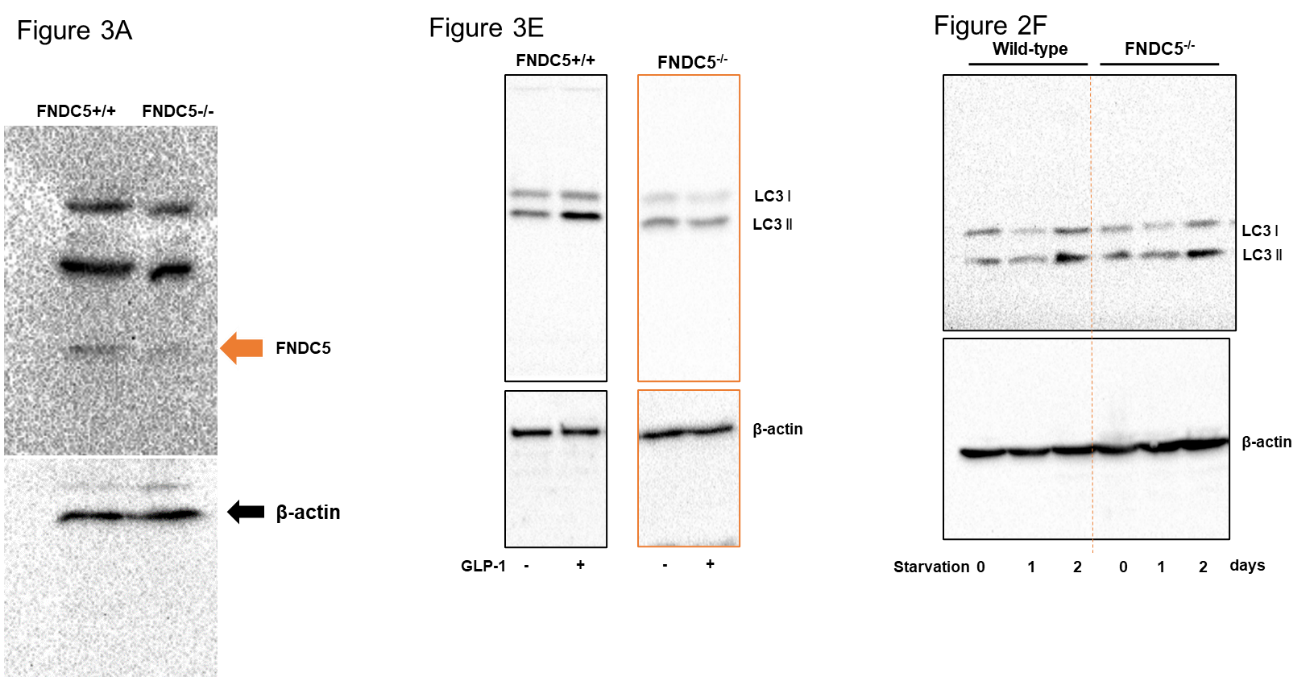


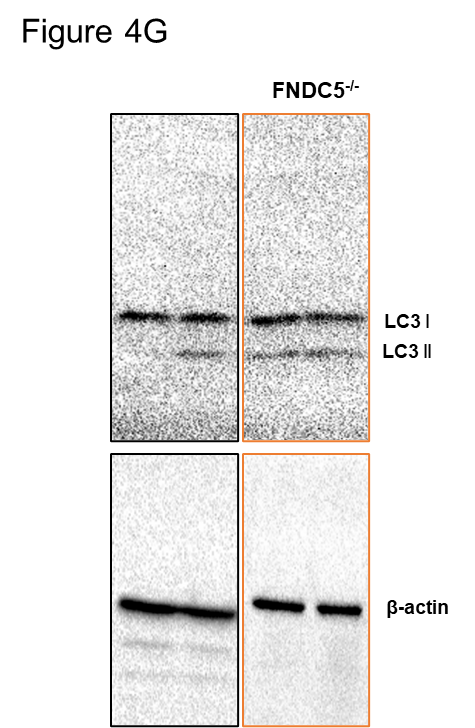


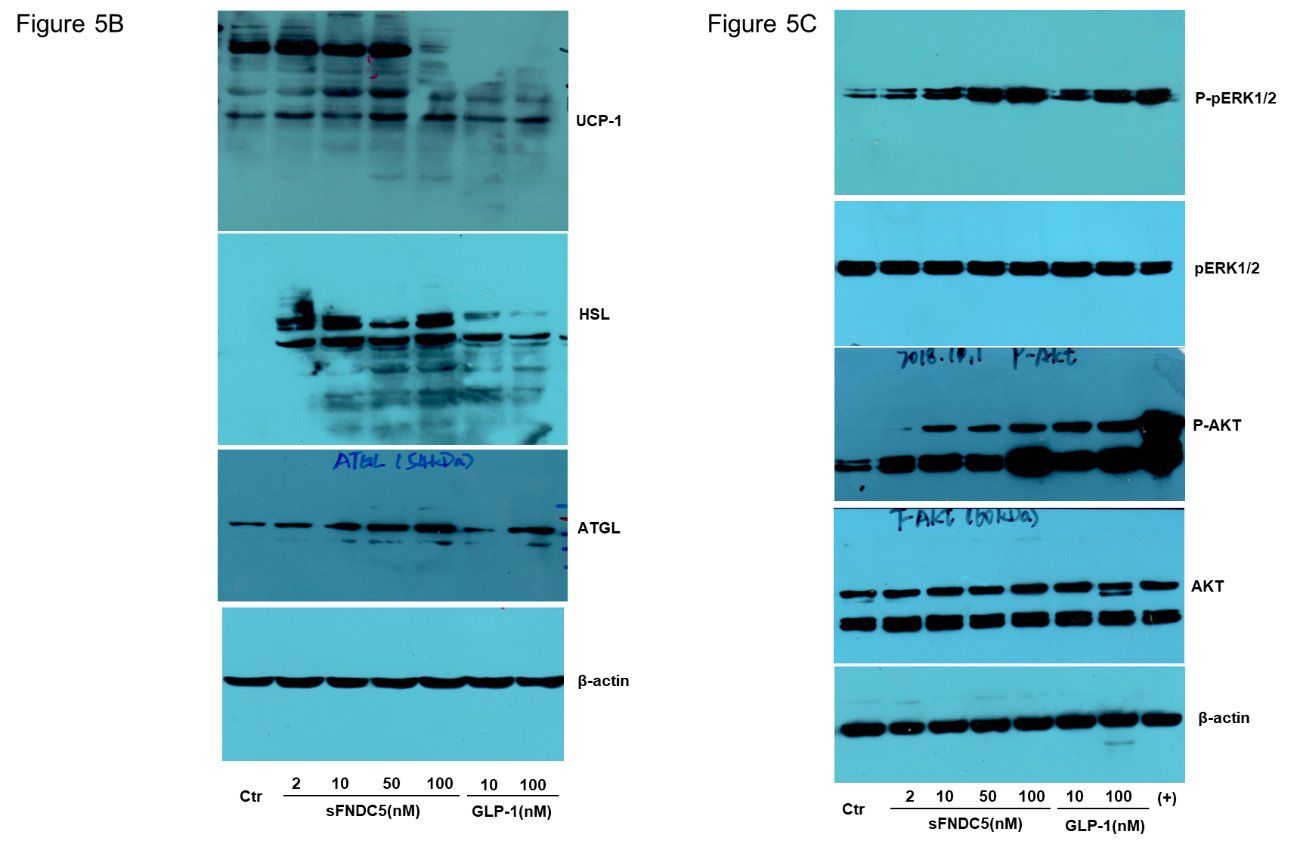


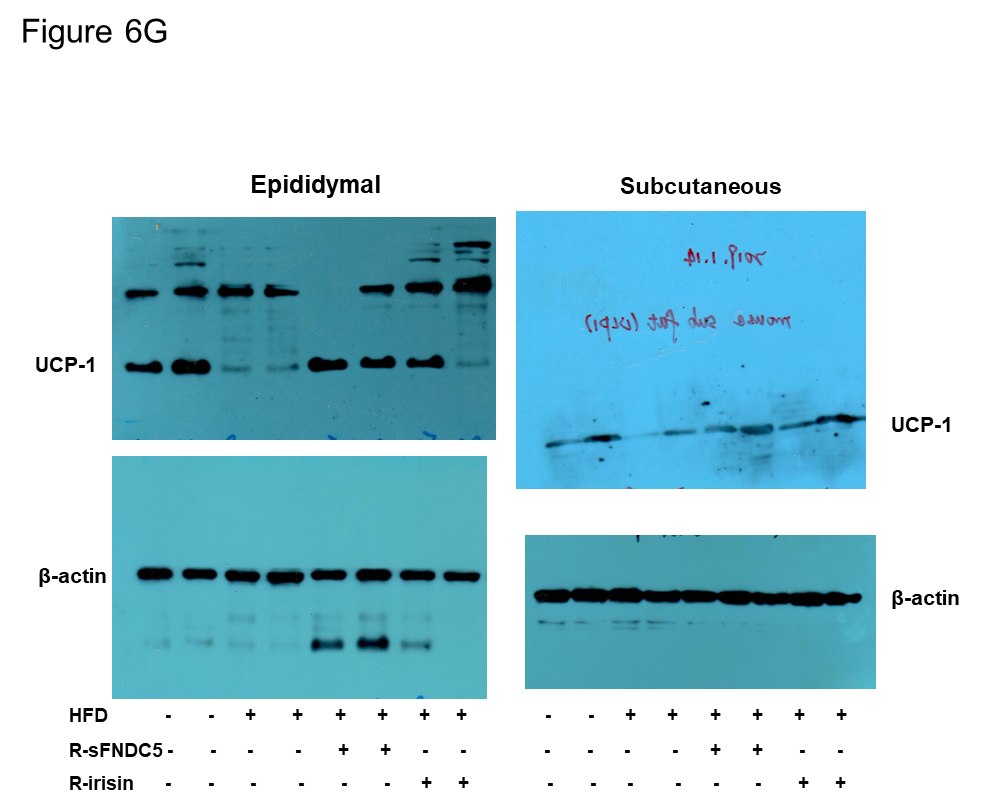

Supplement: Supplementary file 3 [file Table3.DOCX]
